# miR-375-3p suppresses tumorigenesis and partially reverses chemoresistance by targeting YAP1 and SP1 in colorectal cancer cells

**DOI:** 10.18632/aging.102214

**Published:** 2019-09-22

**Authors:** Xueni Xu, Xiaoxiang Chen, Mu Xu, Xiangxiang Liu, Bei Pan, Jian Qin, Tao Xu, Kaixuan Zeng, Yuqin Pan, Bangshun He, Huiling Sun, Li Sun, Shukui Wang

**Affiliations:** 1 School of Medicine, Southeast University, Nanjing 210009, China; 2 General Clinical Research Center, Nanjing First Hospital, Nanjing Medical University, Nanjing 210006, China; 3 Department of Laboratory Medicine, The Second Affiliated Hospital of Nanjing Medical University, Nanjing 210011, China

**Keywords:** miR-375-3p, chemoresistance, YAP1, SP1, Hippo-YAP1 signaling pathway

## Abstract

Clinically, one of the principal factors in the failure of advanced colorectal cancer (CRC) treatment is chemoresistance to 5-fluorouracil (5FU)-based chemotherapy. Although microRNA-375-3p (miR-375) is considered a tumor suppressor in multiple cancers, the mechanism of miR-375 in the regulation of drug resistance in CRC remains unclear. In this study, we investigated the chemosensitivity of miR-375 to 5FU in CRC from biological and clinical aspects. We found that miR-375 was significantly downregulated in CRC tissues and cell lines, and low miR-375 expression was strongly correlated with poor overall survival in CRC patients. Overexpression of miR-375 sensitized CRC cells to a broad spectrum of chemotherapeutic drugs *in vitro* and *in vivo*. Further mechanistic analysis demonstrated that miR-375 enhanced CRC cell sensitivity to 5FU by directly targeting YAP1 and SP1. MiR-375 downregulated YAP1, resulting in reduced expression of the Hippo-YAP1 pathway downstream genes CTGF, cyclin D1 and BIRC5 (also known as survivin). Overall, miR-375 was confirmed as a prospective molecular biomarker in the chemoresistance and prognosis of CRC patients, and the synergy between miR-375 and chemotherapeutic drugs could be a promising therapeutic strategy for CRC patients, especially with chemoresistance.

## INTRODUCTION

Colorectal cancer is the fourth most lethal cancer worldwide, reaching up to almost 700,000 human deaths annually [1]. It is estimated that the incidence of colorectal cancer will rise by 1.67 times by 2040 [2]. In China, there were 376,000 new cases of CRC that caused 191,000 deaths in 2015 [3]. CRC is a remarkably heterogeneous disease governed by multiple molecular pathways [4]. In recent years, chemotherapy has become an advisable option for advanced CRC patients, especially for metastatic CRC [5]. Frustratingly, 50–70% of advanced CRC patients develop tolerance to both classical and biological chemotherapy drugs [6]. Acquired drug resistance poses a major obstacle to curative therapy for CRC patients and leads to poor prognosis [7, 8]. Although therapeutic strategies have made significant progress in recent decades, there is an urgent need to optimize multidrug treatment approaches to efficiently improve anticancer-drug effects as well as to achieve better prognosis and recovery**.**

MicroRNAs (miRNAs), a class of endogenous small noncoding RNA molecules, at the length of ~22 nucleotides, regularly act as repressors of gene expression by inducing target mRNA degradation or transcription inhibition [9]. In recent decades, astounding numbers of miRNAs have been implicated in a variety of boilogical process, including proliferation, apoptosis and metabolism [10–12]. As oncogenes or tumor suppressors, the roles of miRNAs have been extensively explored in numerous cancers, which make them attractive targets for novel therapeutic approaches [13, 14]. Along with the genetic alterations and epigenetic modifications that affect resistance, dysregulation of miRNAs is also involved in tumor cellular variations in chemoresistance [15–18]. However, the aberrant expression of miRNAs can modulate various cancer phenotypes, suggesting that miRNAs may lead to different outcomes of drug resistance in different tumors [19]. Thus, it is a prerequisite to identify the multiple responses of efficient miRNAs in chemoresistance in order to develop accurate multidrug treatments. MiR-375 was reported as an important tumor suppressor that targeted critical oncogenes in the progression of a variety of cancers, such as CRC, hepatocellular carcinoma (HCC), gastric cancer (GC) and cervical cancer. In addition, it was thought to be a potent therapeutic target because it could suppress tumor cell growth *in vitro* and *in vivo* [20]. Interestingly, abundant evidence shows that miR-375 was highly associated with therapeutic sensitivity in hepatocellular carcinoma, prostate cancer and breast cancer [21–23]. Although there is also emerging evidence suggesting the specific suppressive role of miR-375 in colorectal cancer and its crucial function in stratifying patients to preoperative chemoradiation [24, 25], to date, there are insufficient data implicating the underlying mechanism of miR-375 in CRC drug resistance [26]. In particular, data that would shed light on how miR-375 modulates drug resistance by targeting YAP1 in CRC are scarce.

The Hippo signaling pathway is generally acknowledged as a critical player in manipulating the tissue growth, cell proliferation and apoptosis that occur in multiple human cancers. It is composed of mammalian Ste20-like kinases 1/2 (MST1/2), large tumor suppressor 1/2 (LATS1/2), yes-associated protein (YAP, encoded by YAP1) and transcriptional coactivator with PDZ-binding motif (TAZ) [27]. As a vital downstream effector of the Hippo pathway, YAP1 is an essential activator of transcription, as dysregulation of the Hippo pathway triggers YAP/TAZ hyperactivation, which promotes tumorigenesis [28].

In our study, we used two 5FU-resistant cell lines, HCT116/FU and HCT8/FU, and their corresponding parental cell lines, HCT116 and HCT8, to study how miR-375 regulated tolerance to 5FU. We found miR-375 was genetically downregulated in CRC tissues and cells, especially in resistant cell lines, and its low expression level correlated with chemoresistance, malignancy and poor prognosis. Phenotypic experiments showed miR-375 significantly inhibited proliferation, induced apoptosis and had synergistic efficacy with a broad spectrum anticancer drugs, including 5FU *in vitro*. Moreover, we demonstrated for the first time that miR-375 partially reversed 5FU resistance by directly downregulating YAP1 and SP1 in CRC. To conclude, our results verified that miR-375 acted as a potential biomarker for prognosis and therapeutics prediction. Most importantly, it is highly likely that miR-375 will become a novel treatment strategy for CRC in the clinic when combined with chemotherapeutic agents.

## RESULTS

### MiR-375 expression is genetically downregulated in colorectal cancer and negatively correlated with chemoresistance, malignancy and poor prognosis

**Figure 1:**
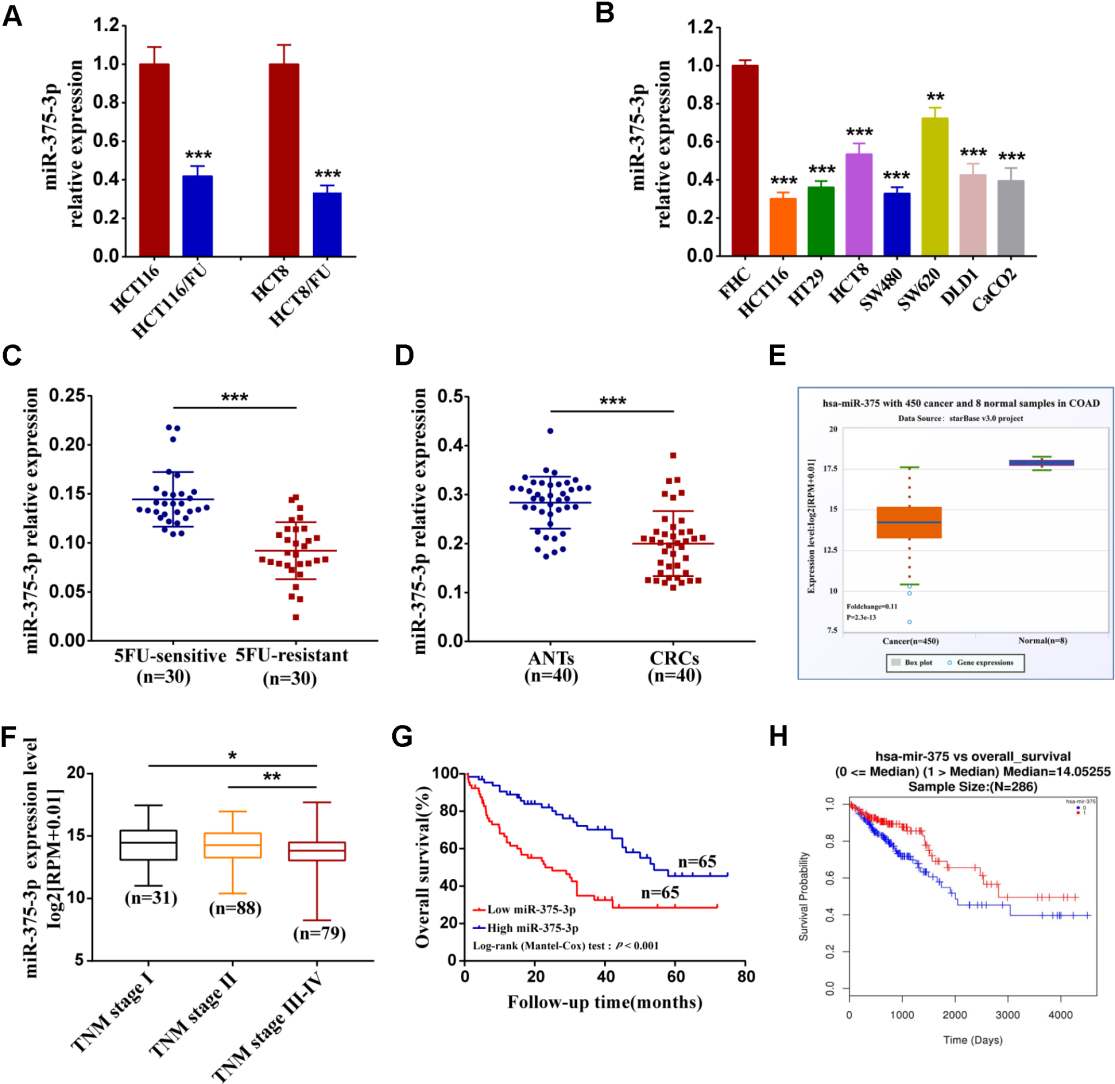
**Downregulation of miR-375-3p associated with chemoresistance, malignancy and poor prognosis.** (**A**) The association of miR-375-3p expression and 5FU-resistance were measured by qRT-PCR in CRC parental cell lines (HCT116, HCT8) and 5FU-resistant cell lines (HCT116/FU, HCT8/FU). (**B**) The miR-375-3p expression in CRC cell lines (HCT116, HT29, HCT8, SW480, SW620, DLD1 and CaCO2) were compared with that in the colonic mucosal epithelial cell (FHC) by qRT-PCR. (**C**) The association of miR-375-3p expression and 5FU-resistance were measured by qRT-PCR in 5FU-sensitive and 5FU-reisistant groups. MiR-375-3p expression was reduced in 5FU-reisistant group. (**D**, **E**) qRT-PCR analysis of miR-375-3p expression in CRC tissues compared with that in adjacent normal tissues from our clinical samples (n = 40, respectively)and Starbase v3.0 database. MiR-375-3p expression was reduced in CRC tissues. (**F**) The association analysis of miR-375-3p expression with TNM stage (I, II, III, IV) in CRC patients from TCGA database are shown. (**G**) Kaplan-Meier survival curves for miR-375-3p expression in associated with overall survival based on our clinical samples (n =130, log-rank test, p < 0.001). (**H**) Kaplan-Meier survival curves for miR-375-3p expression in associated with overall survival from TCGA database. * *P* < 0.05, ** *P* < 0.01 and *** *P* < 0.001.

To demonstrate the association of miR-375 with the response of CRC cells to chemotherapeutics, we established two drug-resistant cell lines, HCT116/FU and HCT8/FU, by long-term culture of their corresponding parental cell lines, HCT116 and HCT8, *in vitro* with escalating 5FU concentrations. The resistance of parental and resistant cell lines to 5FU was examined by treating them with different concentrations of 5FU. As shown from the growth inhibition curves (Supplementary Figure 1A, 1B), the inhibitory rates of resistant cells were significantly decreased compared with their parental cells. The IC50 of 5FU in parental cells was 22.88 ± 0.14μg/ml and 25.59 ± 0.16 μg/ml, respectively, indicating more potency compared with that of resistant cells (146.14±15.06 μg/ml and 140.22±10.40 μg/ml (Supplementary Table 1). To further determine the relationship between miR-375 and chemoresistance, we first analyzed miR-375 expression in parental cell lines HCT116 and HCT8 and established corresponding 5FU-resistant sublines HCT116/FU and HCT8/FU. The results showed that miR-375 was significantly decreased in both of the 5FU-resistant cell lines (Figure 1A). Then, we analyzed miR-375 expression by qRT-PCR and found that miR-375 was lower to different degrees in CRC cell lines than in colonic mucosal epithelial cells (FHC) (Figure 1B). Moreover, clinical samples of patients who relapsed after 5FU-based chemotherapy (the 5FU-resistant group) were compared with those of patients who did not (the 5FU-sensitive group). The results showed that miR-375 expression was much lower in the 5FU-resistant group (n=30) than in the 5FU-sensitive group (n=30), indicating that miR-375 expression was associated negatively with chemoresistance in CRC tissues (Figure 1C). In addition, we compared the expression of miR-375 in 40 paired CRC and their adjacent normal tissues and found that CRC patients generally had downregulated miR-375 in CRC tissues (Figure 1D). Similar results were obtained in 450 CRC and 8 normal specimens downloaded from the Starbase database (Figure 1E). Afterwards, we divided clinical specimens into two groups based on the miR-375 expression value to explore its correlation with clinicopathological variables. A chi-square test showed that the miR-375 expression level was notably correlated with tumor size (*p=*0.038), tumor invasion depth (*p=*0.020) and TNM stage (*p=*0.002) (Table 1). Likewise, the TCGA cohort showed that lower expression of miR-375 was associated with advanced stage (n=198) (Figure 1F). We then investigated the relationship between miR-375 expression and CRC prognosis. Kaplan**–**Meier survival analysis revealed that patients with lower miR-375 levels had a much poorer overall survival than those with higher miR-375 levels (Figure 1G), which was also identified in the TCGA database (Figure 1H). We next performed univariate and multivariate survival analyses to test the correlation of miR-375 expression with overall survival in CRC patients. As shown in Table 2, univariate analysis validated three prognostic factors: miR-375 expression (*p<*0.001), tumor invasion depth (*p* = 0.034) and TNM stage (*p<*0.001). Multivariate survival analysis indicated that miR-375 was an independent prognostic factor (hazard ratio [HR] =2.54; 95% confidence interval [CI] = 1.18–4.33, *P* = 0.001) for CRC patients. These results suggest that miR-375 may play a critical role in the progression and drug resistance of CRC.

**Table 1:** Correlation between miR-375-3p expression and clinicopathological variables of CRC patients (n=130).

**Variables**	**miR-375-3p expression**			***P*-value^1^**
	**Total (n=130)**	**High (n=65)**	**Low (n=65)**	
**Age (years)**				
≤60	58	30	28	0.724
>60	72	35	37	
**Gender**				
Male	63	33	30	0.599
Female	67	32	35	
**Tumor size (cm)**				
≤5	89	50	39	0.038
> 5	41	15	26	
**Tumor invasion depth**				
T1-2	101	56	45	0.020
T3-4	29	9	20	
**Lymph node metastasis**				
N0	50	30	20	0.071
N1-2	80	35	45	
**Distant metastasis**				
M0	74	42	32	0.076
M1-2	56	23	33	
**TNM stage**				
I–II	97	56	41	0.002
III–IV	33	9	24	

^1^Statistical significant results (in bold).

**Table 2:** Univariate and multivariate analysis of clinicopathological parameters for overall survival in 130 CRC patients.

**Variables**	**Univariate analysis**		**Multivariate analysis**	
	**HR (95%CI)**	***P*-value^1^**	**HR (95%CI)**	***P*-value^1^**
miR-375-3p expression (high vs low)	2.48 (1.52-4.05)	**<0.001**	2.54 (1.18-4.33)	**0.001**
Tumor size (≤5cm vs >5cm)	1.28 (0.67-2.51)	0.109		
Tumor invasion depth (T1/2 vs T3/4)	1.31 (1.15-2.91)	**0.034**	1.02(0.85-1.79)	0.548
TNM stage (I/II vs III/IV)	3.69 (2.32-7.87)	**<0.001**	3.35 (1.64-8.03)	**0.001**
Lymph node metastasis (N0 vs N1/2)	0.99 (0.72-1.56)	0.253		
Distant metastasis (M0 vs M1/2)	2.04 (0.95-4.52)	0.086		
Age (≤60 vs >60)	0.64 (0.48-1.34)	0.883		
Gender (male vs female)	0.42 (0.35-1.26)	0.562		

^1^Statistical significant results (in bold)

Abbreviations: CI, confidence interval; HR, hazard ratio.

### MiR-375 inhibits proliferation and chemoresistance and promotes 5FU-induced apoptosis of CRC cells *in vitro*

**Figure 2:**
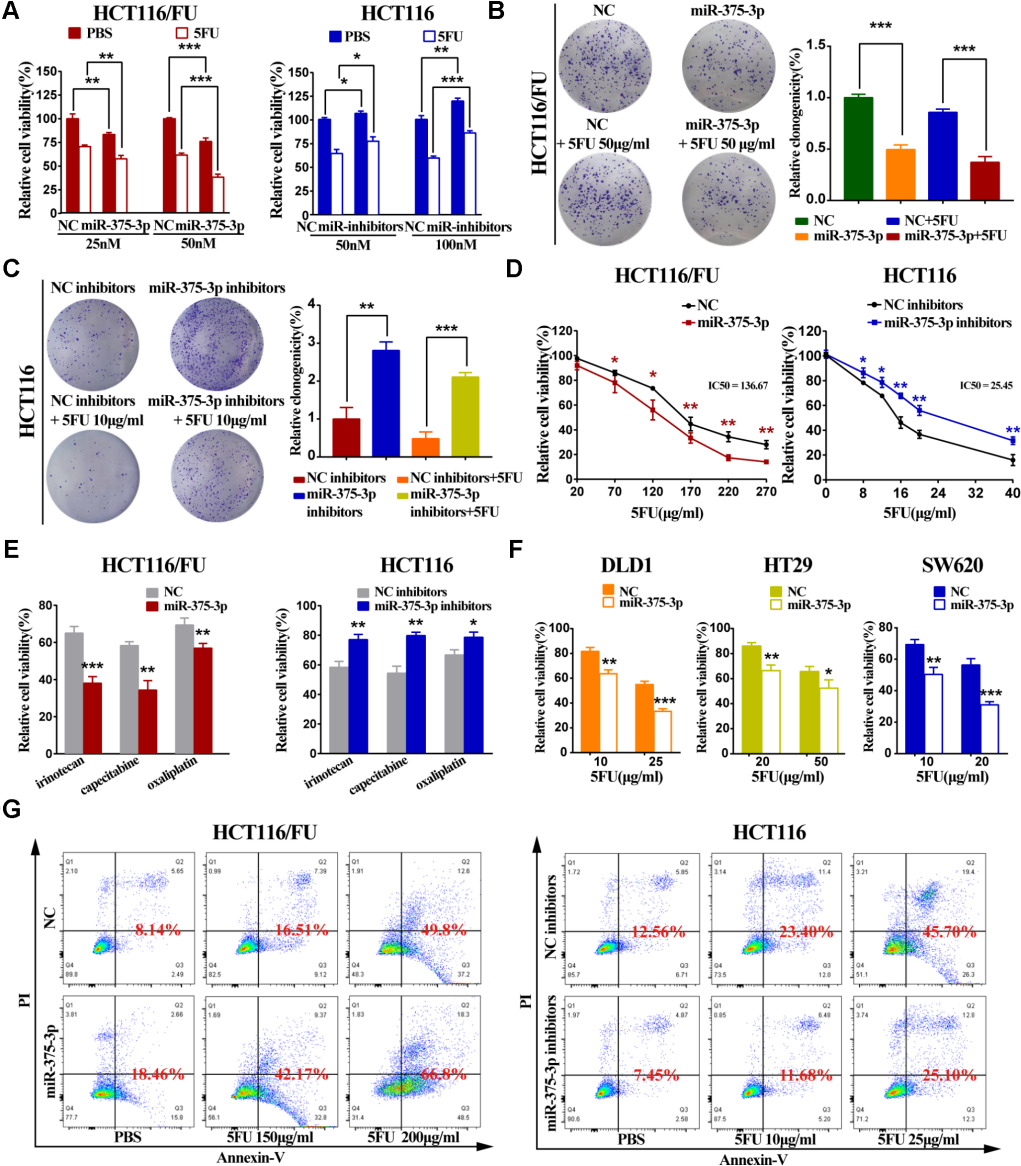
**miR-375-3p inhibits proliferation, chemoresistance and promotes 5FU-induced apoptosis of CRC cells in vitro.** (**A**) Cell proliferation was detected by MTT assay after different concentrations of transfection together with 5FU treatment or not. (Left : HCT116/FU cells transfected with miR-375-3p/NC mimics: 25nM and 50nM; Right : HCT116 cells transfected with miR-375-3p/NC inhibitors : 50nM and 100nM). (**B**, **C**) HCT116/FU and HCT116 cells were respectively transfected with miR-375-3p mimics or inhibitors together with 5FU treatment or not. Colony formation assay were measured to show miR-375-3p inhibited cell growth. (**D**) MTT assay showed overexpression of miR-375-3p increased the sensitivity of HCT116/FU cell lines to 5FU and inhibition of miR-375-3p enhanced the resistance of HCT116 cell lines to 5FU. (**E**) The sensitivity of HCT116/FU(Left) and HCT116(Right) cells to multiple anticancer drugs were measured after transfection. (No transfection of HCT116/FU and HCT116 cell lines were taken as 100% viability, respectively; HCT116/FU cells: oxaliplatin 15μg/ml, irinotecan: 100μg/ml, capecitabine: 40μg/ml; HCT116 cells: oxaliplatin 2.5μg/ml, irinotecan: 18μg/ml, capecitabine: 4μg/ml;). (**F**) The sensitivity of DLD1, HT29 and SW620 CRC cell lines to 5FU were measured after transfection. (No transfection of cell lines were taken as 100% viability, respectively). (**G**) The apoptotic rate of the indicated cells transfected with miR-375-3p mimics or inhibitors, respectively, together with different concentrations of 5FU treatment were detected by flow cytometry analysis. (Left: HCT116/FU cells, concentration groups of 5FU: 150μg/ml and 200μg/ml; Right: HCT116 cells, concentration groups of 5FU: 10μg/ml and 25μg/ml). * *P* < 0.05, ** *P* < 0.01 and *** *P* < 0.001.

Parental cell lines (HCT116 and HCT8) and 5FU-resistant cell lines (HCT116/FU and HCT8/FU) were lipotransfected with miR-375 mimics and inhibitors (Supplementary Figure 1C, 1D). The cell viability experiment demonstrated that overexpression of miR-375 significantly reduced CRC-resistant cell viability, especially when combined with 5FU treatment. While miR-375 inhibitors remarkably increased CRC cell viability and markedly decreased 5FU sensitivity (Figure 2A, Supplementary Figure 2A). A colony formation assay further verified that miR-375 inhibited cell proliferation and promoted 5FU sensitivity, and miR-375 combined with 5FU treatment led to little cell growth in HCT116/FU and HCT8/FU cells. In contrast, miR-375 inhibitors notably increased cell proliferation and enhanced 5FU resistance in HCT116 and HCT8 cells (Figure 2B, 2C, Supplementary Figure 2B, 2C). MTT analysis showed that miR-375 significantly decreased the tolerance of 5FU-resistant cells to 5FU, whereas miR-375 inhibitors distinctly enhanced 5FU resistance in CRC parental cells (Figure 2D, Supplementary Figure 2D). Dramatically, the same observation was found when miR-375 mimics or inhibitors were combined with various anticancer drugs, including capecitabine, oxaliplatin and irinotecan (Figure 2E, Supplementary Figure 2E). The weak drug tolerance level extended to other CRC cells transfected with miR-375, such as DLD1, HT29 and SW620 (Figure 2F). In addition, in CRC parental and resistant cells with 5FU regimens, miR-375 significantly increased the cell apoptosis rate, whereas miR-375 inhibitors decreased the cell apoptosis rate (Figure 2G, Supplementary Figure 2F).

### MiR-375 suppresses tumorigenesis and reverses chemoresistance to 5FU of CRC *in vivo*

**Figure 3:**
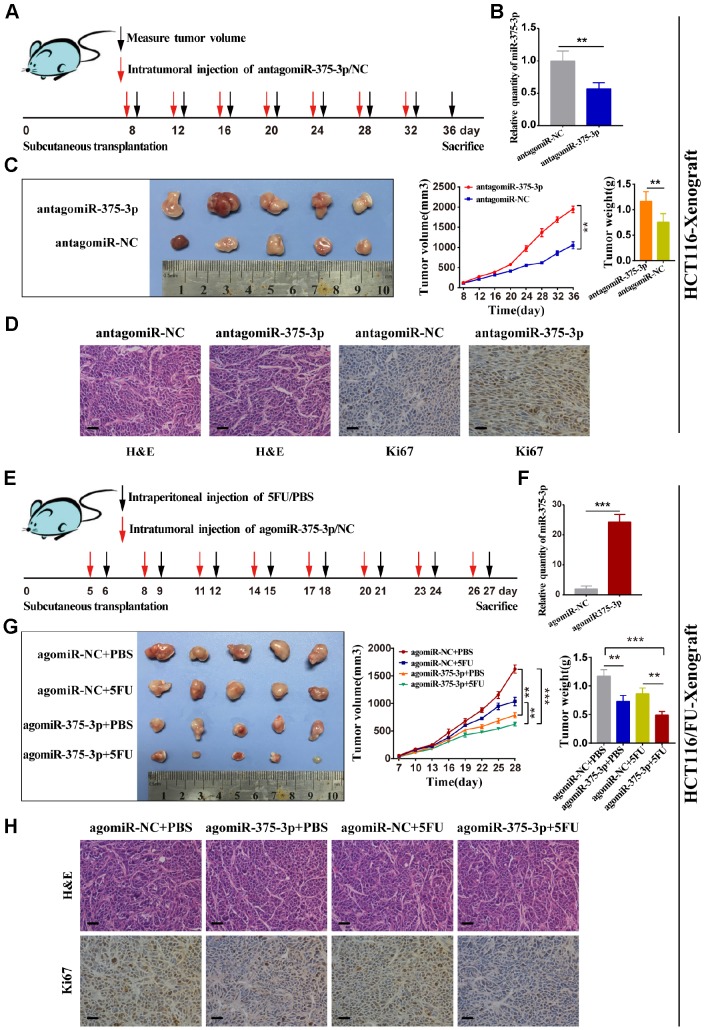
**miR-375-3p suppresses tumorigenesis and reverses chemoresistance with 5FU of CRC *in vivo*.** (**A**) Schematic outline of the treatment of HCT116 cells transfected with antagomiR-375-3p/NC in a subcutaneous tumor model followed by intratumoral injection of antagomiR-375-3p/NC. (**B**) qRT-PCR analysis of miR-375-3p expression in mice xenograft of antagomiR-375-3p/NC groups. (**C**) Representative images of transplanted tumors removed from mice after sacrifice at the 36^th^ day, tumor volume and tumor weight were measured. (**D**) Representative images of hematoxylin and eosin (H&E) staining and Ki67 immunostaining of tumor lumps from two groups. Scale bar=50μm. (**E**) Schematic outline of the treatment of HCT116/FU cells transfected with agomiR-375-3p/NC in a subcutaneous tumor model followed by intratumoral injection of agomiR-375-3p/NC or PBS/5FU. (**F**) qRT-PCR analysis of miR-375-3p expression in mice xenograft of agomiR-375-3p/NC groups. (**G**) Representative images of transplanted tumors removed from mice after sacrifice at the 27^th^ day, tumor volume and tumor weight were measured. (**H**) Representative images of hematoxylin and eosin (H&E) staining and Ki67 immunostaining of tumor lumps from 4 different groups. *P < 0.05, ** *P* < 0.01 and *** *P* < 0.001.

To determine the relationship between miR-375 and therapeutic effects, we used transfected HCT116 cells to transplant subcutaneously into nude mice, followed by subsequent intratumor injection of antagomiR-375 or antagomiR-NC after 7 days (Figure 3A). miR-375 expression was detected in tumors from two groups of nude mice using qRT-PCR. As expected, the miR-375 expression level was notably reduced in tumors from the antagomiR-375 group compared with that in the antagomiR-NC group (Figure 3B). In HCT116-Xenograft, tumor growth was significantly inhibited in the antagomiR-375 group compared with the antagomiR-NC group (Figure 3C). Immunohistochemical (IHC) analysis of tumor tissues collected from the antagomiR-375 group exhibited higher Ki67-positive compared with the control group, which implied that cell proliferation was increased when inhibition of miR-375 (Figure 3D). On the other hand, we conducted agomiR-375 and a relatively low dose of 5FU to explore whether the treatment could result in a synergistic effect *in vivo*. Transfected HCT116/FU cells were inoculated subcutaneously into nude mice to form tumors. All mice were divided into 4 groups with intratumor injections of agomiR-375/NC or 5FU every 3 days (Figure 3E). Consequently, the miR-375 expression level increased by approximately 25-fold compared with the agomiR-NC group (Figure 3F). tumors from the agomiR-375 group were extremely smaller than those from the agomiR-NC group. In addition, tumors combined with agomiR-375 and 5FU treatments presented greater anti-tumor effects than either agomiR-375 or 5FU alone. (Figure 3G). In addition, Ki67 staining showed that Ki67-positive cells were significantly reduced by the combinational therapy (Figure 3H). These results indicated that miR-375 can suppress tumor growth and enhance the chemosensitivity of 5FU *in vivo*.

### YAP1 and SP1 are inversely associated with miR-375 and chemosensitivity

**Figure 4:**
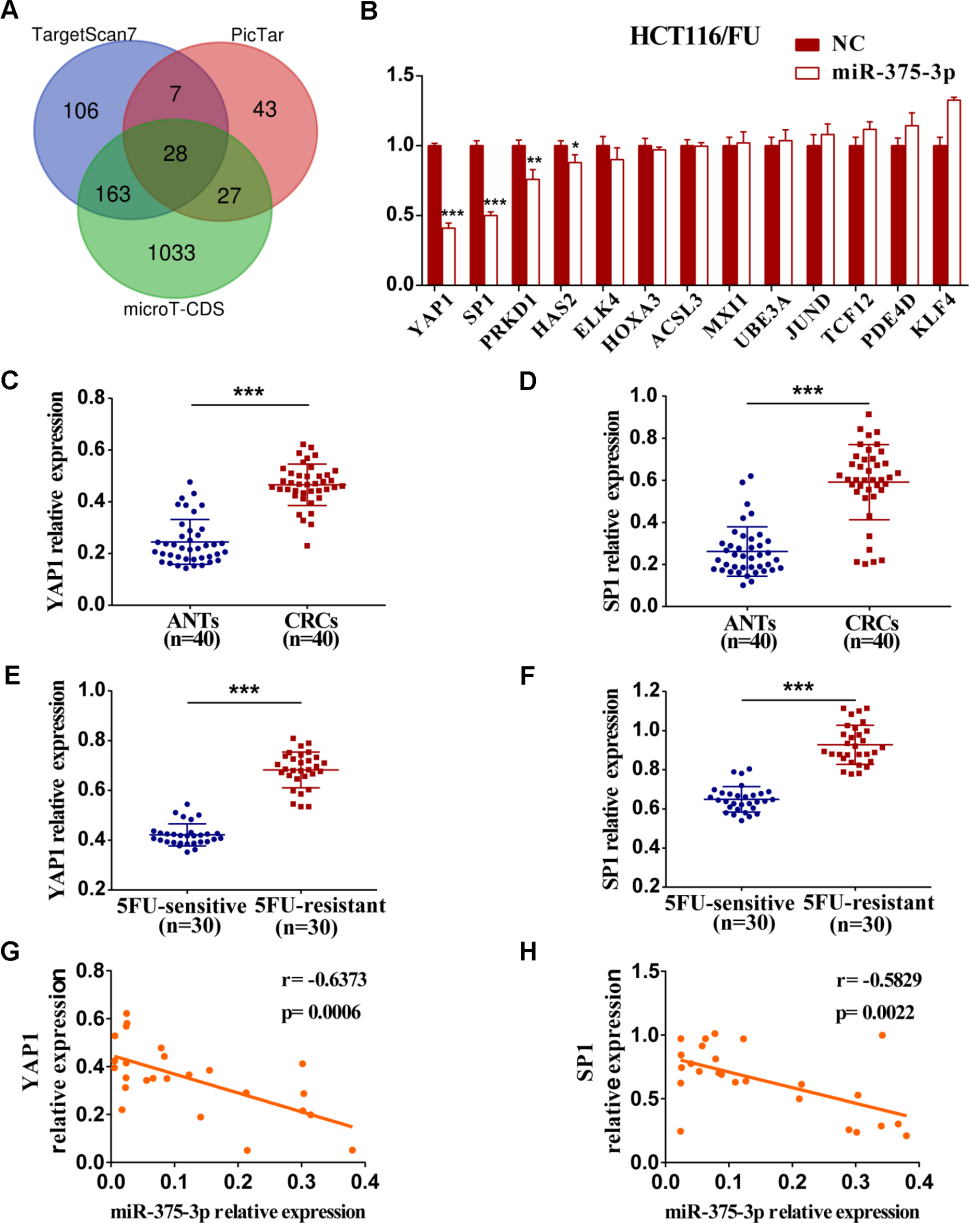
**The expression of YAP1 and SP1 are inversely associated with miR-375-3p and chemosensitivity.** (**A**) Potential target genes of miR-375-3p were predicted in three bioinformatics databases. (**B**) The mRNA expressions of 13 candidate targets were analyzed in HCT116/FU cells transfected with miR-375-3p/NC mimics. (**C**, **D**) The mRNA expression levels of YAP1 and SP1 were analyzed in CRC patients (n=40). (E, F) qRT-PCR analysis of the expressions of YAP1 and SP1 in 5FU-resistant and 5FU-sensitive groups (n=30, respectively). (**G**, **H**) The correlations of miR-375-3p expression and YAP1/SP1 expressions were analyzed in CRC tissues, respectively (n = 25). *P < 0.05, **P < 0.01 and ***P < 0.001.

To identify miR-375 target genes involved in miR-375-mediated suppression of cell proliferation and chemoresistance, three different online miRNA target prediction bioinformatics databases (TargetScan7, PicTar and microT-CDS) were initially applied. Then, 28 potential genes of miR-375 were predicted through integrated analysis of three databases (Figure 4A). Finally, 13 potential candidate target genes that were associated with tumorigenesis and development were screened, and qRT-PCR was carried out later. As shown in Figure 4B and Supplementary Figure 3A, the mRNA expression levels of four genes (YAP1, SP1, PRKD1 and HAS2) among 13 candidate genes were significantly downregulated by miR-375 in HCT116/FU and HCT8/FU cells transfected with miR-375 mimics. Thereafter, we analyzed the mRNA levels of the four genes in CRC clinical specimens and their matched adjacent normal tissues (n=40, respectively) and found that only YAP1 and SP1 were significantly upregulated in CRC tissues (Figure 4C, 4D, Supplementary Figure 3B, 3C). Intriguingly, YAP1 and SP1 expression levels were strikingly higher in 5FU-resistant patients than in 5FU-sensitive patients (n=30, respectively), which revealed a reverse relationship between YAP1/SP1 expression and 5FU sensitivity (n=40, respectively) (Figure 4E, 4F). Moreover, a significantly negative correlation between YAP1 and SP1 expression and miR-375 was found in CRC tissues (Figure 4G, 4H).

### MiR-375 directly targets YAP1 and SP1

**Figure 5:**
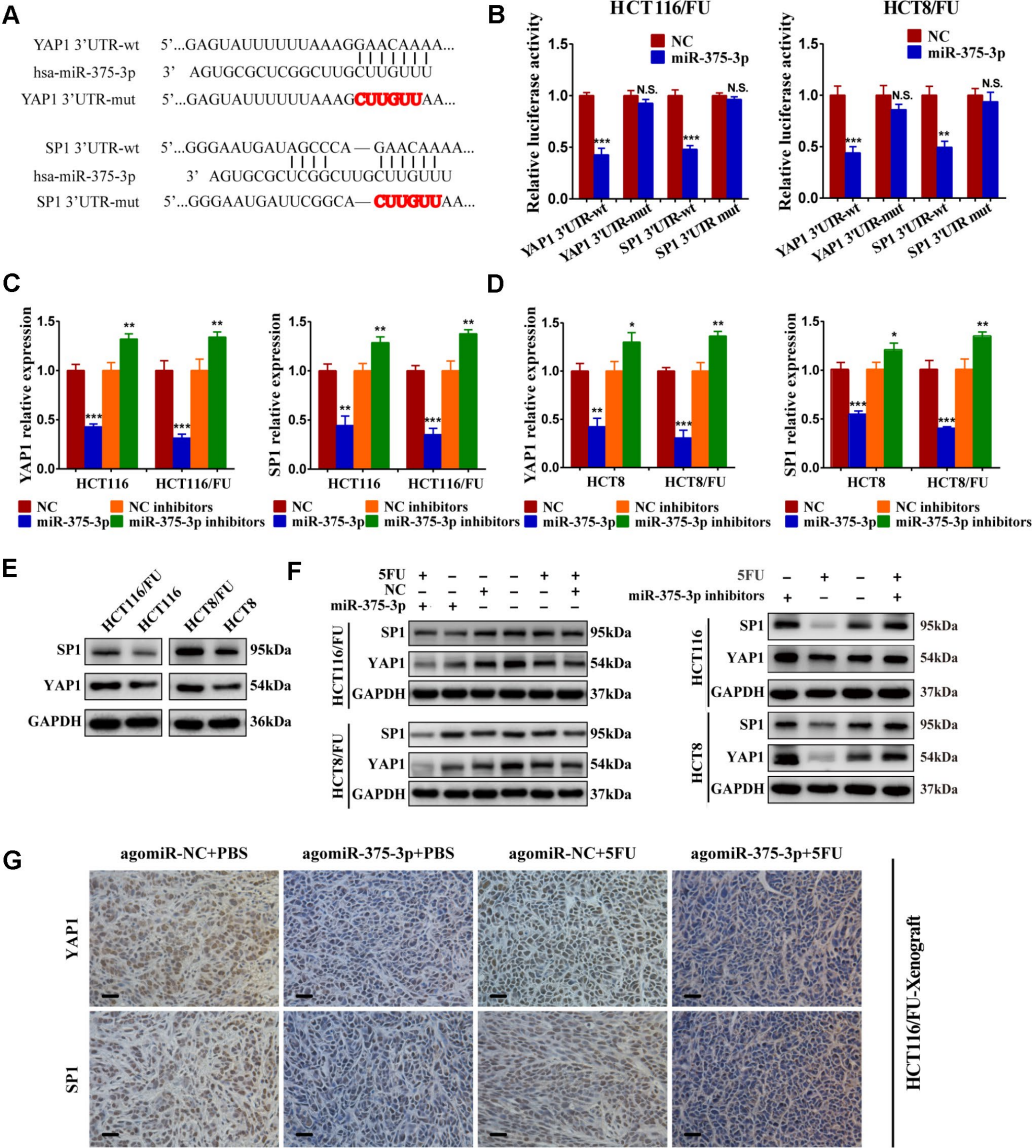
**miR-375-3p directly targets YAP1 and SP1.** (**A**) Predicted binding regions between wild-type (wt) or mutant (mut) 3′-UTRs of YAP1/SP1 and miR-375-3p. The sequences formatted in bold red represent the mutant miR-375-3p binding sites in YAP1 or SP1 3′ UTR. (**B**) Luciferase reporter assay showed the decreased luciferase activity in miR-375-3p-overexpressed cells (HCT116/FU and HCT8/FU) for 3′ UTR wild-type constructs. The luciferase activity was normalized to Renilla luciferase. (**C**, **D**) The mRNA expression levels of YAP1/SP1 in parental cells (HCT116, HCT8) and 5FU-resistant cells (HCT116/FU, HCT8/FU) were analyzed following transfections of miR-375-3p mimics or inhibitors into the four cell lines. (**E**) YAP1 and SP1 protein expression levels were detected by western blot in CRC parental cell lines (HCT116 and HCT8) and 5FU-resistant cell lines (HCT116/FU and HCT8/FU). (**F**) Western blot was performed to analyze the protein expression levels of YAP1 and SP1 not only in 5FU-resistance cell lines (HCT116/FU and HCT8/FU) overexpressed miR-375-3p, but also in CRC parental cell lines (HCT116 and HCT8) inhibited miR-375-3p. **Simultaneously** with 5FU treatment or not. (**G**) Representative images of tumor lumps in HCT116/FU-xenograft that were stained with YAP1 and SP1 by IHC. *P < 0.05, **P < 0.01 and ***P < 0.001.

To verify whether YAP1 and SP1 were direct targets of miR-375, we conducted a dual-luciferase assay. The putative binding sites of miR-375 on the YAP1 and SP1 3’UTR are shown in Figure 5A. After wild-type and mutant-type YAP1 and SP1 3’-UTR sequences were cloned into the luciferase reporter vector, respectively, we found that miR-375 significantly reduced the luciferase activities of the wild-type reporters of YAP1/SP1 3’-UTR both in HCT116/FU and HCT8/FU cells, whereas no obvious decrease was observed in binding the mutant-type reporters of YAP1/SP1 3′-UTR (Figure 5B). The results strongly demonstrated that YAP1 and SP1 were the direct targets of miR-375 in CRC. In particular, overexpression of miR-375 significantly decreased the mRNA expression of YAP1 and SP1, while inhibition of miR-375 remarkably elevated the mRNA expression of YAP1 and SP1 in both parental and 5FU-resistant cell lines (Figure 5C, 5D). Moreover, the total protein expression levels of YAP1 and SP1 were both observably higher in 5FU-resistant cells when compared with their corresponding parental cells (Figure 5E), which further signified that YAP1 and SP1 had a reverse association with miR-375 and 5FU sensitivity. Meanwhile, the total protein expression of YAP1 and SP1 was significantly attenuated by miR-375 in 5FU-resistant cells, whereas notably enhanced by miR-375 inhibitors in parental cells. These effects were more obvious when treated with 5FU. (Figure 5F). Additionally, we subcutaneously injected HCT116/FU and HCT116 cells into BALB/c nude mice and analyzed the levels of YAP1 and SP1 in tumors by IHC assays (Figure 5G, Supplementary Figure 3D), which also confirmed the relationship between YAP1/SP1 and miR-375, drug resistance. These results undeniably indicated that miR-375 negatively regulated YAP1 and SP1 by directly binding YAP1 and SP1 in CRC.

### MiR-375 suppresses cell proliferation and 5FU resistance of CRC cells by repressing YAP1 and SP1

**Figure 6:**
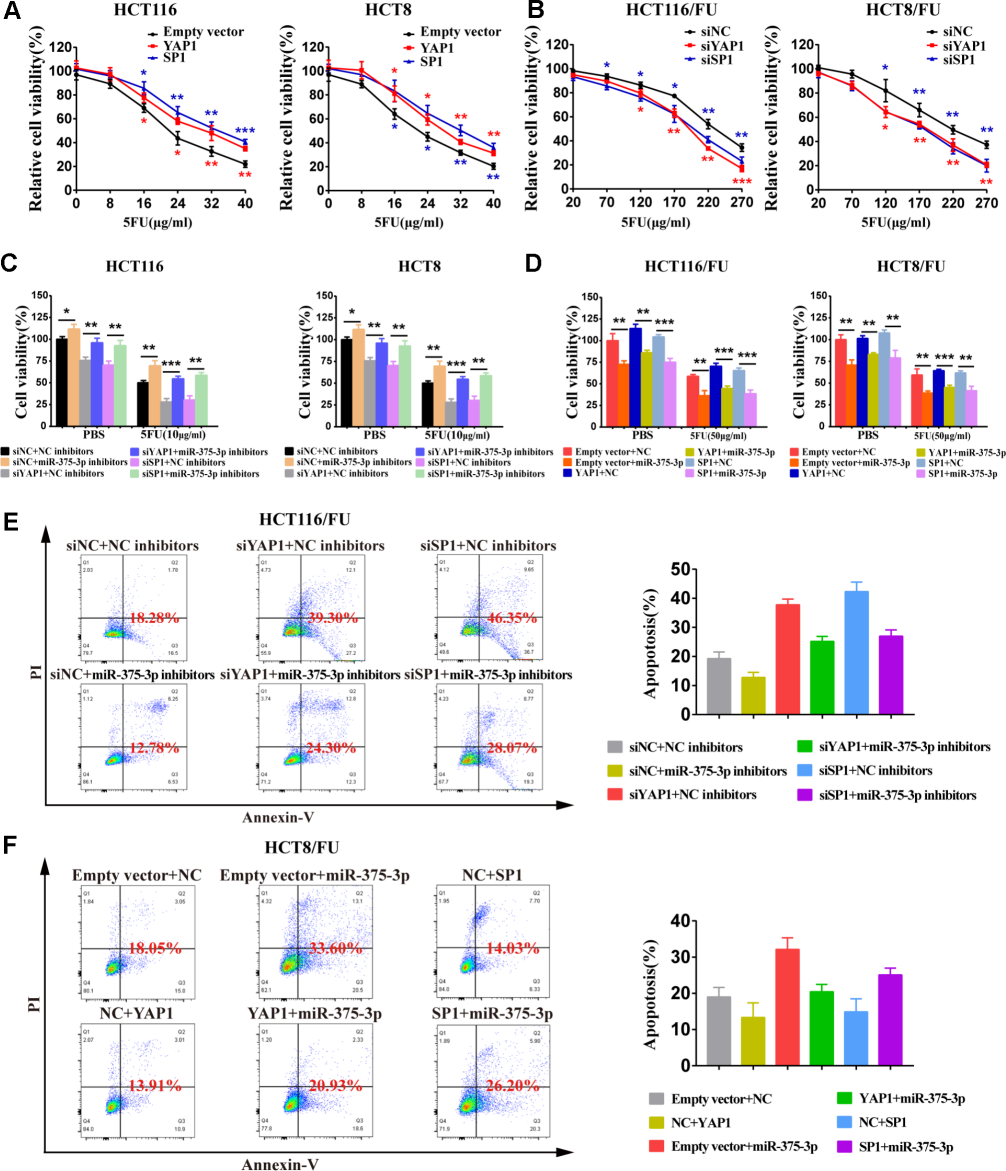
**miR-375-3p suppresses cell proliferation and 5FU resistance of CRC cells by repressing YAP1 and SP1.** (**A**) Overexpression of YAP1 and SP1increased cells resistance to 5FU in CRC parental cell lines, HCT116 and HCT8. (**B**) Depletion of YAP1 and SP1 reversed cells resistance to5FU in CRC resistant cell lines, HCT116/FU and HCT8/FU. (**C**, **D**) Cell viability assays were measured both in parental cell lines (HCT116, HCT8) and 5FU-resistant cell lines (HCT116/FU, HCT8/FU). HCT116 and HCT8 cells co-transfected with miR-375-3p inhibitors and siYAP1 or siSP1 respectively, were treated with 5FU (10μg/ml) or not (PBS). HCT116/FU and HCT8/FU cells co-transfected with miR-375-3p and YAP1 or SP1 overexpressing plasmids respectively, were treated with 5FU (50μg/ml) or not (PBS). (**E**, **F**) The apoptosis rates of HCT116/FU cells with co-transfection of miR-375-3p inhibitors and siYAP1 or siSP1, respectively, and HCT8/FU cells with co-transfection of miR-375-3p and YAP1 or SP1 overexpressing plasmids, respectively, were measured by flow cytometry analysis. Every system treated with low dose of 5FU for 50μg/ml. *P < 0.05, **P < 0.01 and ***P < 0.001.

To determine the relationship between YAP1/SP1 expression and cell proliferation or resistance to 5FU in CRC cells, we knocked down YAP1/SP1 by siYAP1/siSP1 or overexpressed them using special plasmids (Supplementary Figure 3E, 3F). The MTT assay demonstrated that upregulation of YAP1/SP1 significantly induced proliferation and enhanced 5FU resistance in CRC parental cells, whereas downregulation of YAP1/SP1 relatively reduced cell proliferation and drug resistance in CRC 5FU-resistant cells (Figure 6A, 6B). Moreover, when miR-375 inhibitors were cotransfected with siYAP1/siSP1, a cell viability assay indicated that silencing YAP1/SP1 substantially reversed the resistance of miR-375 inhibitors to 5FU with or without drug administration (Figure 6C). In contrast, when miR-375 was cotransfected with YAP1/SP1 into the 5FU-resistant cells, cell viability was obviously decreased by miR-375 with or without 5FU-induced inhibition; nevertheless, the inhibitory effect on cell viability was partially abolished by YAP1/SP1 overexpression (Figure 6D). Moreover, flow cytometry analysis showed that the reduction of 5FU-induced apoptosis by miR-375 inhibitors was entirely reversed by siYAP1 or siSP1 in HCT116/FU cells (Figure 6E), whereas the escalation of 5FU-induced apoptosis by miR-375 was practically undermined by YAP1 or SP1 overexpression in HCT8/FU cells (Figure 6F). Taken together, miR-375 attenuated CRC cell resistance to 5FU by repressing YAP1 and SP1.

### MiR-375 downregulates YAP1 expression via activating the Hippo-YAP1 signaling pathway

As mentioned in Figure 2G, the ectopic expression of miR-375 could significantly promote cell apoptosis in chemoresistant cells, especially when combined with 5FU treatment, and the remedy assay on proliferation and apoptosis implied that YAP1 can reverse the effect of miR-375 in CRC-resistant cells (Figure 6D, 6F). Thus, the potential resistance mechanism of miR-375 associated with YAP1 in CRC cells was further explored in our study.

**Figure 7:**
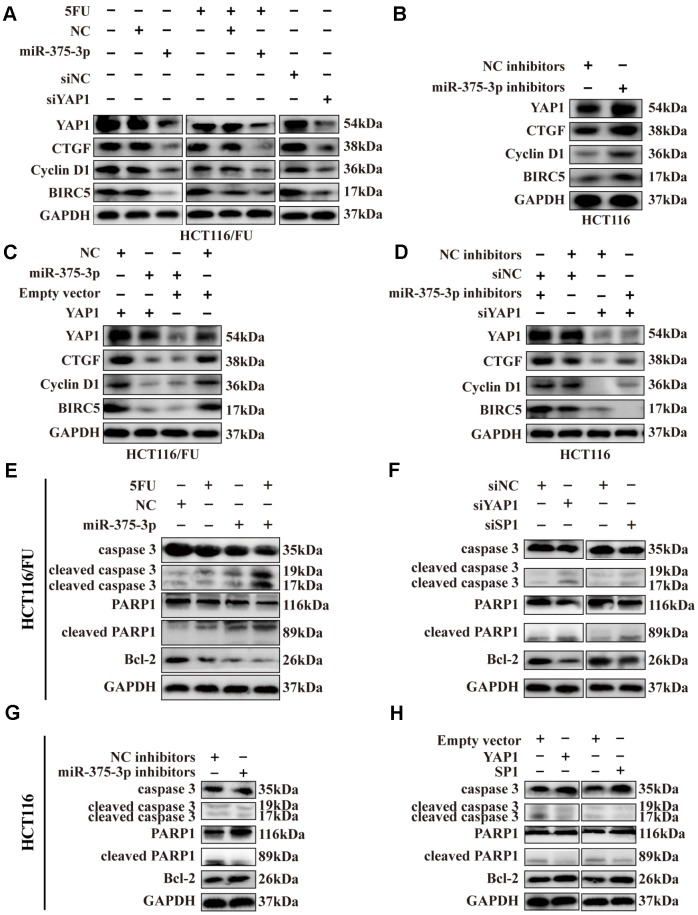
**Expression of SP1, YAP1 and its downstream proteins, apoptosis-related proteins were regulated by miR-375-3p.** (**A**) Transfection of miR-375-3p mimics into HCT116/FU cells with or not with 5FU treatment inhibited the expression of Hippo-YAP1 pathway-associated downstream proteins. Expression of the indicated proteins was detected by western blot. (**B**) Transfection of miR-375-3p inhibitors into HCT116 cells promoted the expression of Hippo-YAP1 pathway-associated downstream proteins. Expression of the indicated proteins was detected by western blot. (**C**) The inhibited effects of miR-375-3p on Hippo-YAP1 pathway-related downstream proteins were abolished by transfection of YAP1 overexpressing plasmid in HCT116/FU cells. (**D**) The promoted effects of miR-375-3p inhibitors on Hippo-YAP1 pathway-related downstream proteins were reversed by transfection of siYAP1 in HCT116 cells. (**E**, **F**) The apoptosis-related proteins expression levels were detected in HCT116/FU cells transfected with miR-375-3p (left) or siYAP1/siSP1 (right). MiR-375-3p and siYAP1 and siSP1 promotes apoptosis in protein level. (**G**, **H**) The apoptosis-related proteins expression levels were detected in HCT116 cells transfected with miR-375-3p inhibitors (left) or YAP1/SP1 overexpressing plasmids (right). MiR-375-3p inhibitors and YAP1 or SP1 inhibits apoptosis in protein level.

Yes-associated protein 1 (YAP1), also known as YAP, is a component of the transcriptional co-activator YAP/TAZ downstream effector inducing target gene expression in the Hippo pathway [29]. According to previous studies [29–31], we selected YAP1 as well as its target downstream proteins related to cell growth, apoptosis and resistance involved in the Hippo pathway, including CTGF, cyclin D1 and BIRC5, and then detected them by western blotting. By overexpressing miR-375 in HCT116/FU cells, we found that total YAP1 expression was significantly downregulated and that its downstream genes CTGF, cyclin D1 and BIRC5 also declined. Surprisingly, compared to nontreatment with 5FU, these protein levels slightly decreased when combined with 5FU cytotoxicity (Figure 7A), which might imply that 5FU inhibited the expression of downstream genes of the Hippo pathway. Similar reductions in these genes were observed in HCT116/FU cells transfected with siYAP1 or siSP1 (Figure 7A). In contrast, miR-375 inhibitors obviously upregulated YAP1 and its downstream targets CTGF, cyclin D1 and BIRC5 in HCT116 cells (Figure 7B). Moreover, miR-375-induced lower levels of protein expression in HCT116/FU cells were observably reversed by YAP1 overexpression (Figure 7C), whereas miR-375 inhibitor-induced higher levels of protein expression in HCT116 cells were moderately counteracted by YAP1 knockdown (Figure 7D).

Meanwhile, apoptosis-related proteins, including cleaved caspase 3 and cleaved PARP1, were increased by miR-375, whereas the expression level of the anti-apoptosis protein Bcl-2 was markedly inhibited in two resistant cells (Figure 7E, Supplementary Figure 4A), similar to the effect of silencing YAP1 or SP1 (Figure 7F, Supplementary Figure 4B). In contrast, miR-375 inhibitors could significantly downregulate the expression of cleaved caspase 3, cleaved PARP1 and elevate the level of Bcl-2 expression in two parental cells (Figure 7G, Supplementary Figure 4C), and a similar result was observed in HCT116 and HCT8 cells overexpressing YAP1 or SP1 vectors (Figure 7H, Supplementary Figure 4D). In addition, IHC revealed that the level of cleaved caspase 3 was significantly increased in xenografts formed from miR-375 overexpression with or without 5FU treatment, while it was decreased from miR-375 inhibition (Supplementary Figure 4E). In summary, miR-375 promoted apoptosis by inhibiting YAP1/SP1 expression. To speak of miR-375-mediated YAP1, miR-375 suppressed YAP1, thereby decreasing the expression of Hippo-YAP1 downstream target genes in CRC cells, which ultimately led to the promotion of apoptosis-related protein expression.

## DISCUSSION

Although chemotherapy has almost become the first-line treatment for most commonly cancer, especially for advanced cancer, statistical data showed that more than 90% of cancer-related deaths attributed to drug resistance, which means chemotherapeutics for curing malignancies often fail from drug resistance of cancers [32]. A previous study suggested that miRNAs participate in the mechanisms of chemotherapy resistance by regulating drug efflux transporters or the cell cycle, drug targets switch and DNA repair [19]. Recently, many studies have also shown that miRNAs are involved in chemotherapy resistance by targeting or influencing specific genes related to cell proliferation, apoptosis and cell cycle [33]. A property of miRNAs that single miRNA usually targets multiple genes and its modulation is tissue-specific may determine one, and the same miRNA has a different even contrary response to drug resistance in different cancers [32]. For instance, miR-214 was notably deregulated in human ovarian cancer and significantly induced cisplatin resistance by targeting PTEN, whereas upregulated miR-214 promoted sensitivity to cisplatin in cervical cancer cells [34, 35]. Recently, accumulating studies reported that miR-195, miR-181a, miR-149 and miR-137 were significantly downregulated, whereas miR-130 was upregulated in adriamycin-resistant breast cancer cells. These results indicated that various miRNAs were involved in adriamycin resistance [36–40]. In our study, we found that miR-375 expression was remarkably decreased in CRC cell lines, especially in 5FU-resistant cells and CRC tissues. The result was consistent with that in the Starbase database. Downregulated miR-375 was markedly related to 5FU resistance in CRC tissues and cell lines, including parental and resistant cells. A recent study has suggested that miR-375 is inhibited by transfection of epithelial-to-mesenchymal transition (EMT), resulting in a reduction of its target thymidylate synthase, which ultimately results in discomfiture of chemoresistance in cancer cells [41]. Analogously, another study indicated that repressive miR-375 reversed tamoxifen simultaneously with EMT-like properties [42]. These findings implied that miR-375 expression brought about partial disadvantages for multidrug resistance. Nevertheless, further discovery presented that miR-375 could reverse the combinational therapy of HCC and doxorubicin resistance through a lipofectin-mediated delivery named L-miR-375/DOX-NPs [22]. The inhibitory effect of miR-375 on doxorubicin in HCC was also identified in another investigation [43]. Moreover, a study showed that miR-375 overexpression increased the chemosensitivity of breast cancer cells [44]. These conflicting results vividly illustrate the complexity of miR-375 and its multifunctional drug effects in different cancers; therefore

As a major tumor suppressor, miR-375 has been extensively mined as a therapeutic target and diagnostic and prognostic biomarker [20]. We speculate that miR-375 may fulfill regulatory drug resistance function in the majority of malignant cancer cells, whereas it seems to be blurry about how miR-375 modulates the therapeutic resistance of CRC cells by targeting YAP1. In our study, we are the first to confirm that miR-375 significantly increased the drug responses of CRC parental and 5FU-resistant cells by activating the Hippo-YAP1 pathway. Similar inhibition by miR-375 of drug resistance was verified using a series of anticancer drugs, including 5FU, oxaliplatin, irinotecan and capecitabine. *In vivo*, a similar effect on chemoresistance was obtained in the HCT116/FU xenograft assay, further affirming miR-375 as a potential target for various kinds of drug resistance. In short, our work offers a network-based perspective to understand how miR-375 acts as a dominant regulator in the reversion of multichemoresistance.

YAP1 was reportedly highly expressed and negatively correlated with overall survival in CRC [45]. Our study demonstrates that YAP1 was the direct target of miR-375 and functioned as a crucial oncogenic participator in CRC. These results are also supported by a previous study [46]. Further evidence suggests a pivotal role for YAP1 in the development of cancer drug resistance. For example, it was reported that exogenous induction of YAP1 induced esophageal cancer cell resistance to 5FU and docetaxel [47]. Another study discovered that YAP1 was a direct target of miR-590-5p in HCC cells, and knockdown of YAP1 reversed the adriamycin-resistant phenotype of HCC cells *in vitro* and *in vivo* [48]. In CRC, a study has suggested that the combination of YAP1 inhibitors with cetuximab can inhibit DDX3-mediated tumor aggressiveness and sensitize CRC to cetuximab [49]. However, we still think the underlying mechanisms of reversion of chemoresistance in CRC require further research. In our study, we uncovered downregulation of YAP1 by miR-375-mediated drug sensitivity via the activation of the Hippo pathway, which resulted in decreases in YAP1 downstream genes related to cell growth or apoptosis: CTGF, cyclin D1 and BIRC5. CTGF has been reported to promote CRC progression by exerting effects on EMT and angiogenesis [50]. Cyclin D1 is a critical factor that regulates G1-S cell cycle progression by forming complexes with Cdk4 and Cdk6 [51]. BIRC5 belongs to the family of apoptosis inhibitor proteins and can repress cell apoptosis by inhibiting caspases 9, 3 and 7 [52]. The Hippo signaling pathway has recently gained extensive attention because it plays a leading role in cancer cell proliferation, apoptosis, differentiation and tumorigenesis [53]. It is known that activation of the Hippo-YAP1 pathway leaves YAP1 phosphorylated and in the cytoplasm, which in turn impairs YAP-dependent transcription in the nucleus, suppressing tumorigenesis [27]. Hippo-YAP1 signaling is essential for tumorigenesis and tumor progression in both epidermal and dermal cells. The latest research found that it was responsible for epidermal cells’ dominant cell growth and differentiation, while dermal fibroblasts respond to YAP1 signaling by sensing the physical cellular environments and promoting ECM deposition and remodeling [28]. Regarding drug resistance and the Hippo-YAP1 pathway, a previous study indicated that the Hippo-YAP1 pathway played a crucial role in 5FU resistance in colorectal cancer [54]. A recent study has further defined a compelling mechanism by which high cell density activated the Hippo-YAP1 pathway, leading to high levels of CDA and gemcitabine efflux pumps, which in turn reduced intracellular concentration of gemcitabine and ultimately came to gemcitabine resistance [55]. Based on these theories and our research, we hypothesized that low expression of miR-375 upregulates YAP1 and activates the Hippo-YAP1 pathway, which in turn leads to 5FU efflux pumps exporting more 5FU, leaving a decreased intracellular 5FU concentration and leading to 5FU resistance in CRC.

Specificity protein 1 (SP1) is a well-known transcription factor that serves as a novel target for CRC therapy since it is involved in CRC progression and development [56]. SP1 has been well documented in predicting the poor prognosis of CRC [57, 58]. Recent research provided evidence that SP1 participated in the activity of Oct4 suppressing PTEN, leading to the activation of AKT signaling and drug resistance in lung cancer [59]. Another study revealed that SP1 could modulate drug resistance by regulating surviving (BIRC5) expression through the ERK-MSK MAPK pathway [60]. Although research has defined SP1 as a direct target of miR-375 in CRC [61], there are scarce data regarding the functions of SP1 underlying miR-375-mediated drug resistance in CRC. In the current study, we confirmed that SP1 modulated miR-375-mediated 5FU resistance in CRC cells. Taken together, the results confirm that directly targeting YAP1/SP1 and miR-375 would be a potential therapeutic strategy to reverse multiple chemoresistance in CRC through the regulation of the Hippo signaling pathway.

**Figure 8:**
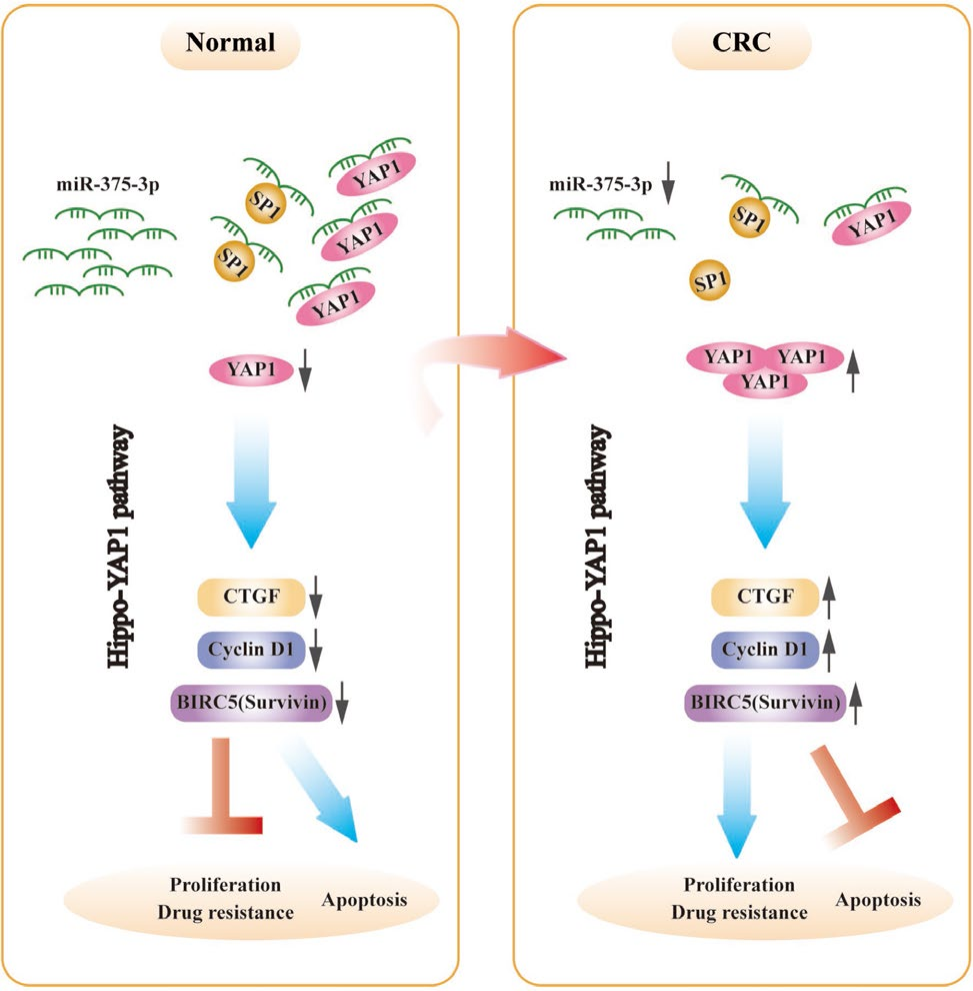
**The schematic model of miR-375-3p as a role in regulating functions in CRC cells.** In CRC, miR-375-3p is frequently downregulated and directly targets YAP1 and SP1 in CRC. Low expression of miR-375-3p leads to the release of YAP1 and SP1, resulting in chemoresistance and tumorigenesis. The mechanism of YAP1 mediated by miR-375-3p promoting drug resistance and proliferation is that release of YAP1 leads to YAP1 increase in CRC cells, thereby activates the downstream genes of Hippo-YAP1 pathway, causing CTGF, Cyclin D1 and BIRC5 (Survivin) upregulate, which ultimately promotes CRC proliferation and drug resistance and inhibits apoptosis.

A genetic regulatory pattern in CRC is diagrammed in Figure 8. We have identified that miR-375 is correlated with cell proliferation, apoptosis and drug resistance. Mechanistic analysis shows that miR-375 is significantly downregulated in CRC, which enhances drug resistance and cell proliferation and inhibits apoptosis by releasing YAP1 and SP1, and the activation of the Hippo-YAP1 pathway upregulates YAP1 expression. Then, YAP1/TAZ coactivators combine with TEADs to activate transcription [29], triggering Hippo-YAP1 downstream gene expression (CTGF, cyclin D1 and BIRC5). Furthermore, miR-375 has been identified as a molecular biomarker associated with malignancy, poor overall survival and chemotherapeutic drug resistance in CRC. Overall, the implication of this study is that a promising therapeutic strategy would be highly expected to restore miR-375 levels or deliver miR-375 to the body, synchronously combined with chemotherapeutic agents such as 5FU. We offer a novel insight into the synergistic therapeutic pathways through which CRC patients who suffer from chemoresistance might attain maximum benefit.

## MATERIALS AND METHODS

### Dual-luciferase reporter assay

Based on the wild-type and mutant 3’UTRs of target genes (YAP1/ SP1) binding to miR-375-3p (Figure 5A), the wt and mut vectors were constructed and subcloned into the pmirGLO-basic luciferase reporter (GeneCreat, China). In 1×10^4^ HCT116/FU or HCT8/FU cells per well in a 96-well plate cultured for 12 h, 50 ng of the wt or mut vector was cotransfected with either miR-375-3p mimics or NC mimics (50 nM) into the two cells using Lipofectamine 2000 according to the manufacturer’s protocol (Invitrogen, CA, USA). After 48 h of transfection, cells were harvested, and luciferase activity was measured by the dual-luciferase reporter assay system (Promega, WI, USA). Renilla luciferase activity was used as an internal reference. Each transfectant was performed in triplicate.

### Cell viability assay and colony formation

The short-term effects of anticancer drugs on cell growth were measured by a cell viability assay. First, 1.5×10^4^ cells/ml were harvested and seeded into 96-well plates for 12 h of cultivation. Then, drug regimens were added into designated columns. After 72 h, cell viability assays were detected by MTT assays. “Relative cell viability” = the viability of cells in drug-containing medium/the viability of cells in drug-free medium. “Relative cell viability” was further fitted to a dose-response curve to estimate the IC50 by GraphPad Prism 7 software. The long-term effects of anticancer drugs on cell growth were assessed with a colony formation assay. After 36 h of transfection, a density of 200 cells/well was plated into 6-well plates and incubated for 48 h. Afterwards, different concentrations of 5FU (10 μg/ml and 50 μg/ml) were added to the designated wells. Cells were cultured for approximately 14 days. The colonies were fixed with methanol for 5 minutes and stained with 0.1% crystal violet for 15 minutes at room temperature. Finally, colonies were photographed and counted using ImageJ k 1.45.

### Analysis of apoptosis

For apoptosis analysis, cells were seeded into 6-well plates (1.5×10^5^ cells/well) for 12 h incubation. MiR-375-3p mimics and NC mimics were transfected into HCT116/FU and HCT8/FU cells, while miR-375-3p inhibitors and NC inhibitors were transfected into HCT116 and HCT8 cells. After 12 h of transfection, each group was treated with PBS and different concentrations of 5FU (inhibitors: 10 μg/ml, 25 μg/ml; mimics: 150 μg/ml, 200 μg/ml) for 48 h. In addition, HCT116/FU cells were cotransfected with miR-375-3p inhibitors and YAP1 or SP1 small interfering RNAs (siYAP1 or siSP1, respectively) (miR-375-3p inhibitors 100 nM; siYAP1/siSP1 50 nM). Meanwhile, HCT8/FU cells were cotransfected with miR-375-3p and YAP1- or SP1-expressing plasmids (miR-375-3p 50 nM; YAP1/SP1 50 nM). After 12 h of transfection, the cell culture medium was changed, and the cells were cultured for 48 h. Cell apoptosis was detected with the Annexin V-FITC/Propidium Iodide (PI) Apoptosis Detection Kit (BD, Biosciences, CA, USA) and analyzed by fluorescence-activated cell sorting using a FACScan (BD Biosciences, San Jose, CA, USA).

### *In vivo* tumorigenicity study

All animal experiments were approved by the Animal Care Committee of Nanjing First Hospital, Nanjing Medial University (acceptance No. SYXK 20160006). For HCT116 xenograft animal models, 4-week-old male BALB/c nude mice were randomly divided into two groups (n=5 per group). HCT116 cells transfected with antagomiR-375-3p or antagomiR-NC (a total of 5 × 10^6^ cells in 0.2 ml PBS) were subcutaneously injected into the right flank region of each nude mouse. After 8 days of tumor formation, 2 nmol antagomiR-375-3p/NC was injected intratumorally every 4 days for seven cycles. For HCT116/FU xenograft animal models, HCT116/FU cells transfected with agomiR-375-3p or agomiR-NC (a total of 5 × 10^6^ cells in 0.2 ml PBS) were subcutaneously injected into the right flank region of each nude mouse. Five days later, all types of xenografts were randomly divided into four groups and treated with (I) agomiR-NC+PBS (10 mg/kg); (II) agomiR-375-3p (2 nmol per mouse)+PBS, (III) agomiR-NC+5FU (10 mg/kg), or (IV) agomiR-375-3p+5FU. AgomiR-375-3p/NC and 5FU were administered via intratumoral injection. The corresponding treatment regimens were repeated every 3 days for eight cycles. Xenografts were measured every 3 days with digital calipers, and tumor volumes were calculated using the formula V =1/2 (L × W^2^). The curve of tumor growth was made based on tumor volume and corresponding time (days) after treatment. Tumor weights were measured using an electronic scale after mice were sacrificed. AgomiR-375-3p, antagomiR-375-3p and their negative controls agomiR-NC and antagomiR-NC were obtained from GenePharma (Shanghai, China).

## Supplementary Material

Supplementary Methods

Supplementary Figures

Supplementary Tables
