# Supplementary material for: miR-375-3p suppresses tumorigenesis and partially reverses chemoresistance by targeting YAP1 and SP1 in colorectal cancer cells
Source: Aging (Albany NY). 2019 Sep 22;11(18):7357–85. doi: 10.18632/aging.102214 (PMC6781994; doi:10.18632/aging.102214)
Supplement: Supplementary Methods [file aging-11-102214-s001.pdf]

SUPPLEMENTARY MATERIALS

Growth inhibition assay

Cell Counting kit-8 (CCK-8) assay (KeyGEN BioTECH, China) was used to assess the inhibition growth of parental and resistant cell lines in response to 5FU (0, 5, 10, 20, 40, 80, 120, 160 and 200 μg/ml). Briefly, parental and resistant cell lines (1.0×10^4 cells/well) were seeded into 96-well plates in 100 μl culture medium/well for 24h. Drugs diluted with culture medium were added to each well for 24h co-incubation. At the end of the treatment, the culture medium was replaced with fresh culture medium containing 10% CCK-8 reagent and the reaction was allowed to proceed for 2 h. Absorbance was measured at the wavelength of 450 nm. All assays were performed as three replicates and the results were indicated as a percentage compared with the corresponding control group.

RNA-extraction and quantitative real-time polymerase chain reaction (qRT-PCR)

Total RNA was extracted from tissues and cells using Trizol LS reagent (Invitrogen, CA, USA). For miR375-3p, reverse real-time quantitative polymerase chain reaction(qRT-PCR) was measured using Hairpin-*it*^{TM} microRNA Normalization Real-Time PCR Quantitation Kit (GenePharma, Shanghai, China),with U6 snRNA as an internal control. For mRNAs, total RNA was first reverse transcribed into cDNA and then quantitative-PCR (q-PCR) was conducted with SYBR® Premix Ex TaqTM Kit (Takara, Otsu, Japan). GAPDH served as the reference gene. All PCR reactions were performed in triplicate. The relative expression of miR375-3p and mRNAs were quantified in $2^{-\Delta\Delta Ct}$ method. The primer sequences of miR375-3p and mRNAs used are listed in Supplementary Table 1.

siRNA design, plasmid construct and cell transfection

The mature miR375-3p, miR-375-3p inhibitors and their corresponding negative controls (NC, NC inhibitors), as well as small interference RNA against YAP1 and SP1 (siYAP1, siSP1) and their corresponding negative controls (siNC) were synthesized by RiboBio (Guangzhou, China). pcDNA3.1-YAP1/SP1 overexpression plasmids and the empty vectors were obtained from Gene Create (Wuhan, China). Transfection was performed by different concentrations of 25nM or 50nM miR-375-3p mimics, 50nM or 100nM miR-375-3p inhibitors and 100nM siRNAs, 50nM over-expressed plasmids using Lipofectamine^{TM} 2000 (Invitrogen, CA, USA) following the manufacturer's instruction. Cells were treated with drug regimens after transfection for 24 h when necessary. SiRNAs sequences were listed in Supplementary Table 2.

Western blot

All cultured cells were collected and lysed with RIPA lysis Buffer. Then, total protein concentration was detected with BCA Protein Assay Kitt (KGP902, KeyGEN BioTECH, Nanjing, China) followed by a quarter of 5 × SDS loading buffer was added. The same amounts of denatured protein were fractionated by 10% sodium dodecyl sulfate polyacrylamide gel electrophoresis and then transferred to polyvinylidene difluoride membranes(Millipore, Schwalbach, Germany). The membranes were blocked with 5% skim milk powder, then incubated with primary antibodies at 4 °C with gentle shaking overnight., subsequently, covered with secondary antibodies at room temperature for 1 hour. The bands were visualized with an enhanced chemiluminescence kit (MA0186, Meilunbio, Dalian, China). Assay was repeated at least three times. The antibodies used in assay were listed in Supplementary Table 3.

Immunohistochemistry (IHC)

IHC assay was performed on formalin-fixed, paraffin-embedded sections of xenograft tumor tissues. The complex was visualized using DAKO Envision System (DAKO, Carpinteria, CA) in accordance with manufacturer's guidelines. All sections were calculated with H-score approach and validated by two pathologists. The staining intensity was classified into 4 categories: nostaining=0,

weakstaining=1, moderate staining=2, strong staining=3. The proportion of positively stained tumor cells were quantified by Image J k 1.45. Then an H-score was calculated using the following formula[1]: [1 × (% cells 1+) + 2 × (% cells 2+) + 3 × (% cells 3+)] × 100. The antibodies used were listed in Supplementary Table 3.

Clinical samples and cells culture

The paraffin-embedded of primary CRC samples and paired adjacent normal tissues were obtained from patients who had undergone surgery at Affiliated Nanjing First Hospital of Nanjing Medical University (Nanjing, China). The fresh CRC tissues and paired adjacent normal tissues were also from Affiliated Nanjing First Hospital of Nanjing Medical University.

Surgically removed tissues were quickly frozen in liquid nitrogen until RNA extraction. The clinical characteristics of 80 samples were retrieved from clinical databases and from the original pathology. The effect of chemotherapy on the tumors was assessed as the three-dimensional volume reduction rate or tumor response rate. The clinical characteristics of CRC patients received 5FU-based chemotherapy were listed in Supplementary Table 5. This study was approved by the ethics committee of Nanjing First Hospital, and informed consent was obtained from each participant prior to surgery.

The colonic mucosal epithelial cell (FHC) and CRC cell lines (HCT116, HCT8, HT29, SW480, SW620, DLD1 and CaCO2) were purchased from American Type Culture Collection (Manassas, VA, USA) and had been tested and authenticated through STR (Short Tandem Repeat) method. HCT116, HT29, SW480, SW620, CaCO2 cells were cultured in Dulbecco's modified Eagle's medium (DMEM) supplemented with 10% fetal bovine serum (FBS) (Gibco, Vienna, Austria). HCT8, DLD1 cells were cultured in Roswell Park Memorial Institute (RPMI) 1640 medium supplemented with 10% fetal bovine serum (Gibco, Vienna, Austria). All cells in these two medium were placed in a humidified atmosphere of 5% CO_2 at 37 °C.

Bioinformatics analysis

Gene expression profiles of 450 CRC samples was downloaded from StarBase v3.0 (http://starbase.sysu.edu.cn). TNM stage with miR-375-3p expressions and overall survival informations were obtained from TCGA data portal (https://genome-cancer.ucsc.edu/). The target genes of miR-375-3p were screened out from TargetScan7.1 (http://www.targetscan.org), PicTar (https://pictar.mdc-berlin.de/cgi-bin/PicTar_vertebrate.cgih) and microT-CDS (http://www.microrna.gr/microT-CDS).

Statistical analysis

All data are expressed as mean ± SD (standard deviation) from independent experiments in triplicate. Statistical analysis was produced by GraphPad Prism5.0 (GraphPad Software, USA) or SPSS 20.0 (IBM, USA). The Student's t-test was used to analyze the data differences between two groups, and one-way ANOVA were employed to evaluate the data differences among three independent groups. Kaplan–Meier method was performed to evaluate the overall survival rate and analyzed by log rank test. The correlations analysis used Pearson's correlation coefficients. A chi-square test was used to analyze the different distribution of clinicpathological variables. Cox proportional hazards models were adopted to analyze the univariate and multivariate analysis *, *P < 0.05;* **, *P < 0.01;* *** *P < 0.001. P values < 0.05* were perceived as statistically significant. All P-values were two sides.

REFERENCE

1. Xu M, Chen X, Lin K, Zeng K, Liu X, Pan B, Xu X, Xu T, Hu X, Sun L, He B, Pan Y, Sun H, et al. The long noncoding RNA SNHG1 regulates colorectal cancer cell growth through interactions with EZH2 and miR-154-5p. Mol Cancer. 2018; 17:141. https://doi.org/10.1186/s12943-018-0894-x
