# Supplementary material for: miR-375-3p suppresses tumorigenesis and partially reverses chemoresistance by targeting YAP1 and SP1 in colorectal cancer cells
Source: Aging (Albany NY). 2019 Sep 22;11(18):7357–85. doi: 10.18632/aging.102214 (PMC6781994; doi:10.18632/aging.102214)
Supplement: Supplementary Figures [file aging-11-102214-s003.pdf]

SUPPLEMENTARY FIGURES

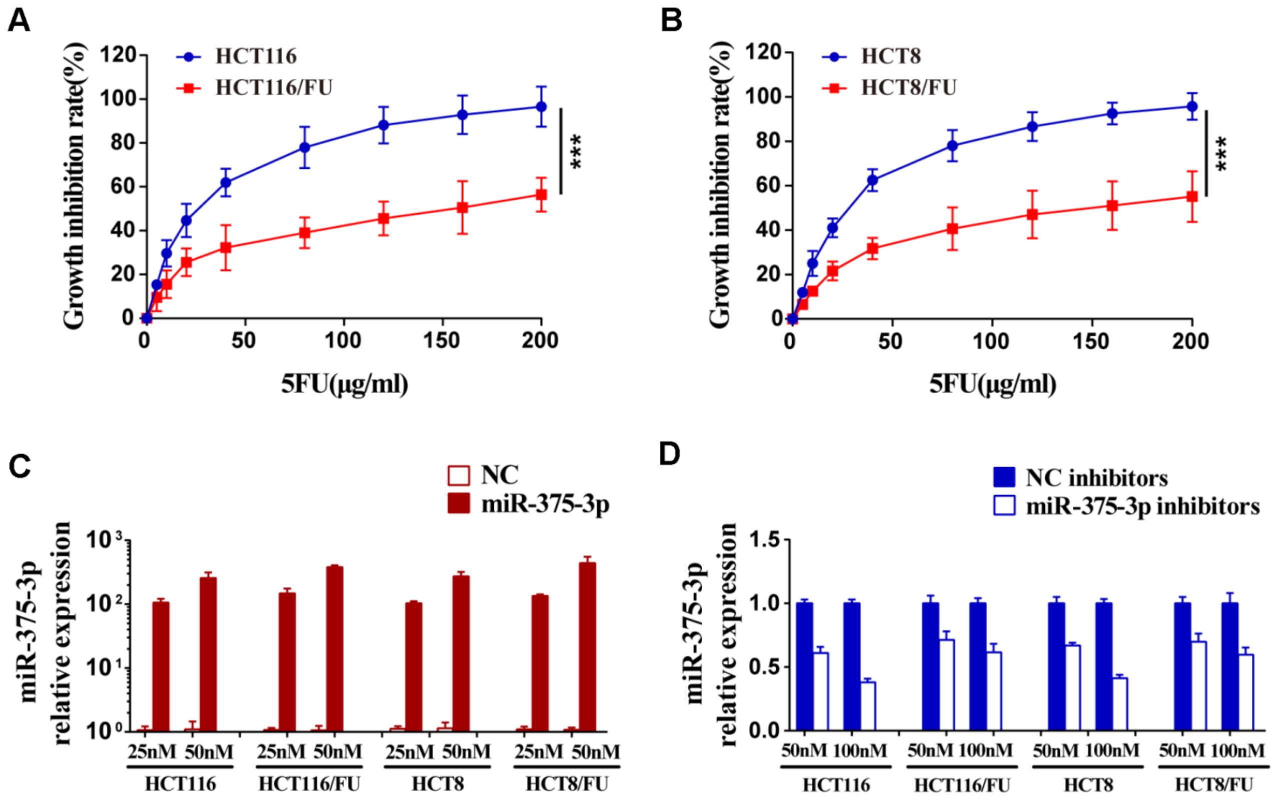

Supplementary Figure 1. Quantitative PCR analysis in CRC. (**A**, **B**) Cell Counting kit-8 (CCK-8) assay was used to assess the inhibition growth of parental and resistant cell lines (HCT116, HCT116/FU and HCT8, HCT8/FU) in response to 5FU (0, 5, 10, 20, 40, 80, 120, 160 and 200 μg/ml). (**C**) Quantitative PCR analysis. HCT116, HCT116/FU, HCT8 and HCT8/FU cells were transfected with miR-375-3p mimics (miR-375-3p) or NC mimics (NC) (25 or 50 nM). The expression levels of miR-375-3p were measured. (**D**) Quantitative PCR analysis. HCT116, HCT116/FU, HCT8 and HCT8/FU cells were transfected with miR-375-3p inhibitors or NC inhibitors (50 or 100 nM). The expression levels of miR-375-3p were measured. *$P < 0.05$, **$P < 0.01$ and ***$P < 0.001$. All assays were performed in triplicate and values represent the mean of three independent experiments.

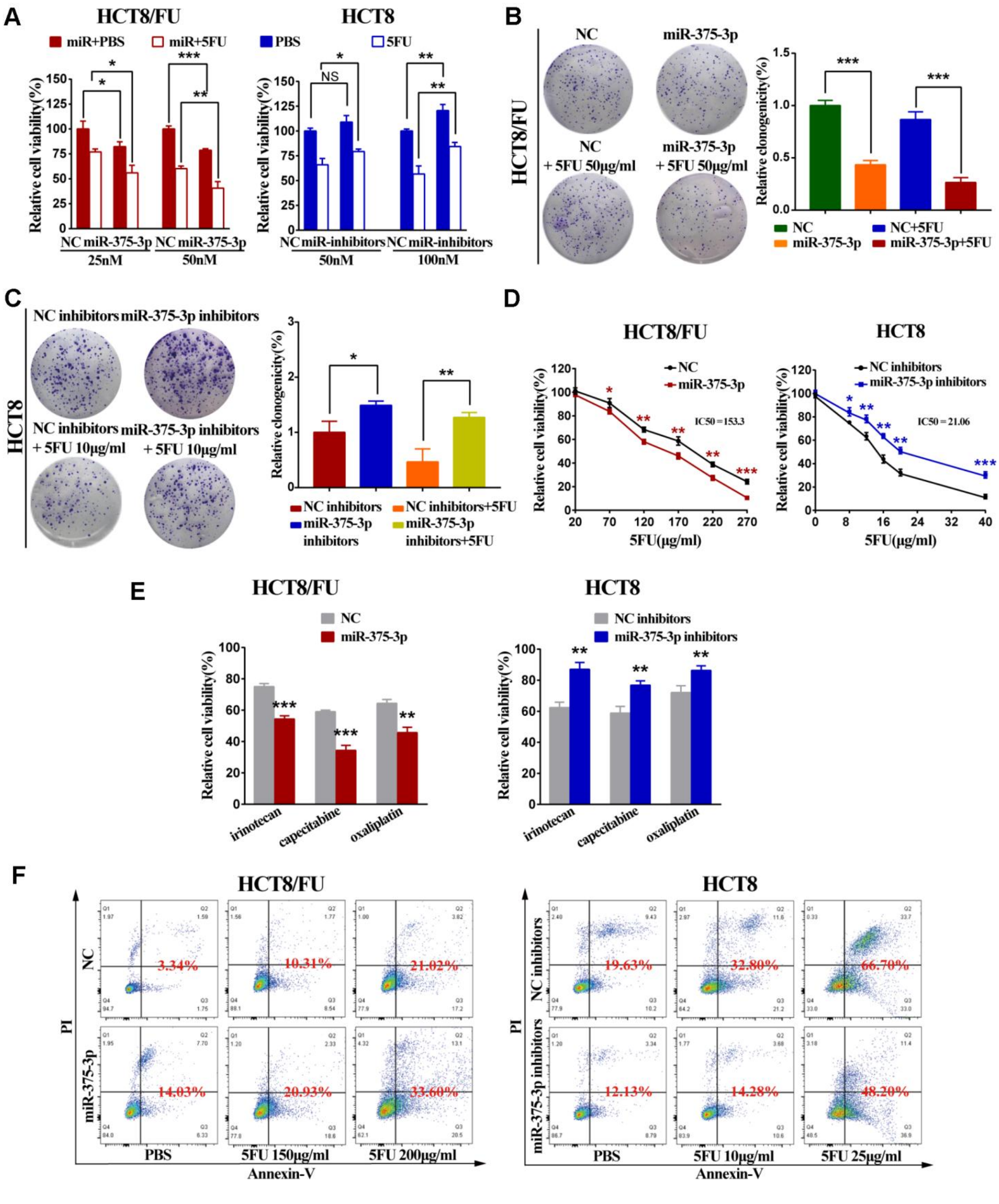

Supplementary Figure 2. miR-375-3p inhibits proliferation, chemoresistance and promotes 5FU-induced apoptosis of CRC cells *in vitro*. (**A**–**D**) HCT8 and HCT8/FU cells were transfected with miR-375-3p mimics or inhibitors, respectively. cell viability assay, colony formation assay and MTT assay were measured. (**E**) The sensitivity of HCT8/FU(left) and HCT8(right) cells to multiple anticancer drugs were measured. (HCT8/FU cells: oxaliplatin 15μg/ml, irinotecan: 100μg/ml, capecitabine: 40μg/ml; HCT8 cells: oxaliplatin 2.5μg/ml, irinotecan: 18μg/ml, capecitabine: 4μg/ml). (**F**) The apoptosis assay of HCT8/FU and HCT8 cells with special treatments were performed by flow cytometry analysis. (Left: HCT8/FU cells, concentration groups of 5FU: 150μg/ml and 200μg/ml; Right: HCT8 cells, concentration groups of 5FU: 10μg/ml and 25μg/ml). *$P < 0.05$, **$P < 0.01$ and ***$P < 0.001$.

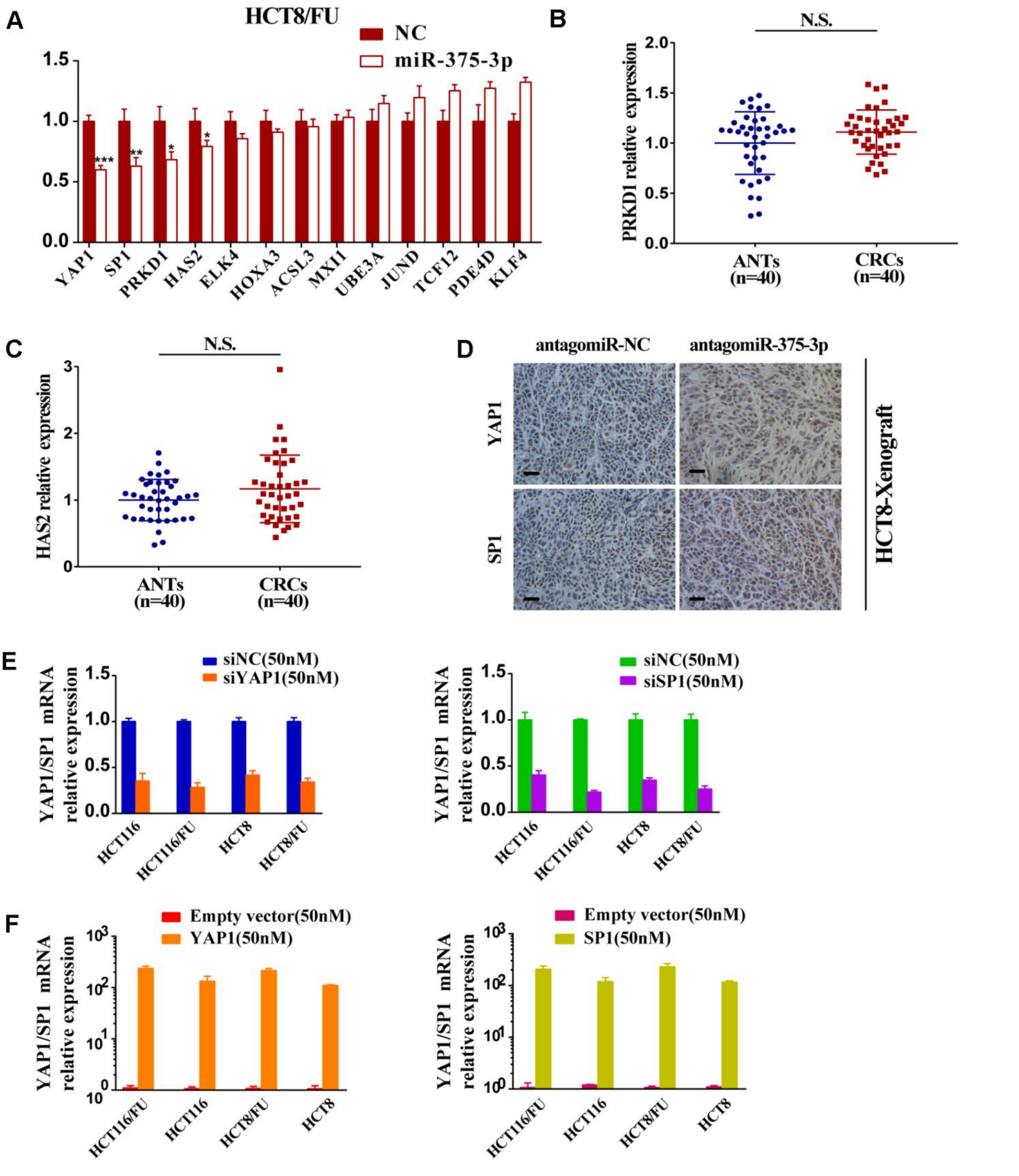

Supplementary Figure 3. Analysis of miR-375 candidate target genes in CRC, including quantitative PCR and IHC. (**A**) The mRNA expressions of 13 candidate targets were analyzed in HCT8/FU cells transfected with miR-375-3p/NC mimics. (**B**, **C**) Quantitative PCR analysis. The mRNA expression levels of PRKD1 and HAS2 in tumor tissues were compared with that in normal tissues in CRC patients (n = 40, respectively). (**D**) Representative images of tumor samples in HCT8-xenograft that were stained with YAP1 and SP1 by IHC. Scale bar=50μm. (**E**) Quantitative PCR analysis. HCT116, HCT116/FU, HCT8 and HCT8/FU cells were transfected withYAP1/SP1 siRNA (50 nM) or siNC (50 nM). The expression levels of YAP1/SP1 were measured. (**F**) Quantitative PCR analysis. HCT116, HCT116/FU, HCT8 and HCT8/FU cells were transfected withYAP1/SP1 overexpression plasmids (50 nM) or corresponding empty vectors (50 nM). The expression levels of YAP1/SP1 were measured. $^{*}P < 0.05$, $^{**}P < 0.01$ and $^{***}P < 0.001$.

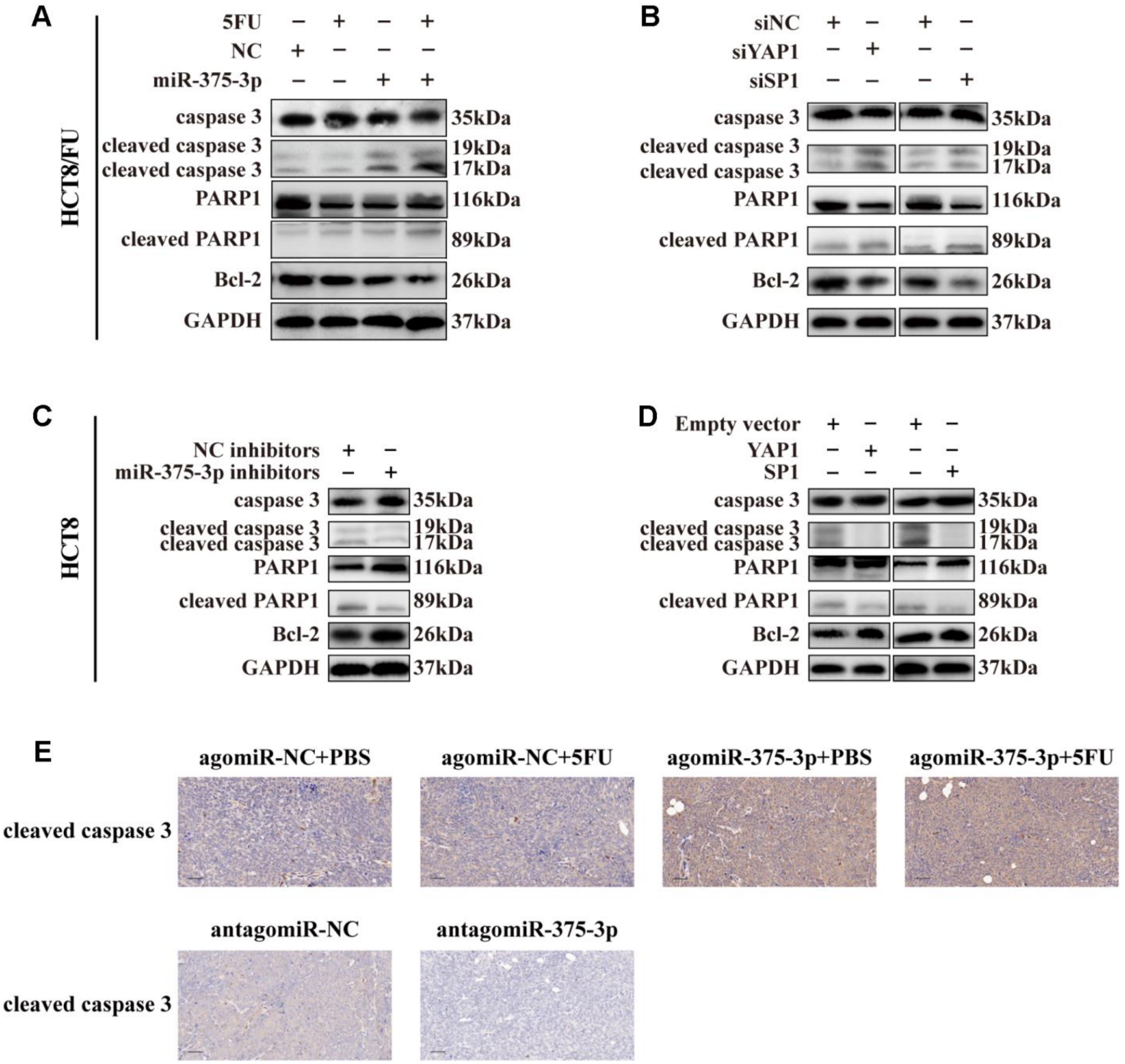

Supplementary Figure 4. Western blot results and growth inhibitory curve analysis. (**A–D**) The expression levels of apoptosis-related proteins in HCT8/FU and HCT8 cells were measured by western blotting according to specific treatments. (**E**) Representative images of cleaved caspase 3 immunostaining of tumor lumps from different groups. Scale bar=50μm.
