# Supplementary material for: miR-375-3p suppresses tumorigenesis and partially reverses chemoresistance by targeting YAP1 and SP1 in colorectal cancer cells
Source: Aging (Albany NY). 2019 Sep 22;11(18):7357–85. doi: 10.18632/aging.102214 (PMC6781994; doi:10.18632/aging.102214)
Supplement: Supplementary Tables [file aging-11-102214-s002.pdf]

## SUPPLEMENTARY TABLES

**Supplementary Table 1. The sensitivity of parental cell lines (HCT116, HCT8) and 5FU-resistant cell lines (HCT116/FU, HCT8/FU) to anticancer drugs.**

| Drug | HCT116 IC50* (μg/ml) | HCT8 IC50* (μg /ml) | HCT116/FU IC50* (μg /ml) | HCT8/FU IC50* (μg /ml) |
|---|---|---|---|---|
| **5- Fluoruouracil** | 22.88 ± 0.14 | 25.59 ± 0.16 | 146.14±15.06 | 140.22±10.40 |
| **Irinotecan** | 41.26 ± 2.82 | 43.17 ± 1.11 | 259.40±2.43 | 267.03+1.56 |
| **Capecitabine** | 9.10 ± 0.22 | 8.44 ± 1.01 | 98.01±5.89 | 102.00±3.23 |
| **Oxaliplatin** | 6.60 ± 1.10 | 8.00 ± 1.99 | 32.07±7.58 | 33.62±3.02 |

IC50*, The IC50 values were defined as the concentration of cells inhibiting growth at 50%.

**Supplementary Table 2. Sequences for primers used in this study.**

| Name | Sequence |
|---|---|
| **q-PCR primers** | |
| has-mir-375-3p-F | CTTACTATCCGTTTGTTCGTTCG |
| has-mir-375-3p-R | TATGGTTGTTCTCGTCTCTGTGTC |
| U6-F | CAGCACATATACTAAAATTGGAACG |
| U6-R | ACGAATTTGCGTGTCATCC |
| YAP1-F | TAGCCCTGCGTAGCCAGTTA |
| YAP1-R | TCATGCTTAGTCCACTGTCTGT |
| SP1-F | TGGCAGCAGTACCAATGGC |
| SP1-R | CCAGGTAGTCCTGTCAGAACTT |
| PRKD1-F | TTCTCCCACCTCAGGTCATC |
| PRKD1-R | TGCCAGAGCACATAACGAAG |
| HAS2-F | GCCTCATCTGTGGAGATGGT |
| HAS2-R | ATGCACTGAACACACCCAAA |
| ELK4-F | CTGGTGCCAAGACCTCTAGC |
| ELK4-R | TCGGCTGGATTCTCAGTCTT |
| HOXA3-F | TCATTTAAGAGCGCCTGGACA |
| HOXA3-R | GAGCTGTCGTAGTAGGTCGC |
| ACSL3-F | TGCTTTCCGAAGCTGCTATT |
| ACSL3-R | AAGGCATCTGTCACCAGACC |
| MXI1-F | TGGCTACGCCTCTTCATTCC |
| MXI1-R | CTGTTGGCAGTGCTGGTGTT |
| UBE3A-F | CCATGGGAAAATGTACATCCA |
| UBE3A-R | TTTTTCAGCTGGTTGTGGAGG |
| JUND-F | TCAAGACCCTCAAAAGCCAGAA |
| JUND-R | TTGACGTGGCTGAGGACTTTC |
| TCF12-F | GCTCGACGCTAGGATCTGAC |
| TCF12-R | GCTTTCCACGACGGTGAC |
| PDE4D-F | AGGGTCTGGGCTGATTCTC |
| PDE4D-R | GTTGATGGATGGTTGGTTGC |
| KLF4-F | AAGCCAAAGAGG GGAAGACG |
| KLF4-R | GTGCCTGGTCAGTTCATCTGAG |
| GAPDH-F | GAAGGTGAAGGTCGGAGTC |
| GAPDH-R | GAAGATGGTGATGGGATTTC |

**Supplementary Table 3. Sequences for siRNAs used in this study.**

| Name | Sequence |
|---|---|
| **siRNAs** | |
| siYAP1 # 1(sense) | GGUGAUACUAUCAACCAAA |
| siYAP1# 1(antisense) | CCACUAUGAUAGUUGGUUU |
| siYAP1 # 2(sense) | GAUGUCUCAGGAAUUGAGA |
| siYAP1# 2(antisense) | CUACAGAGUCCUUAACUCU |
| siYAP1 # 3(sense) | AACUCGGCUUCAGGUCCUC |
| siYAP1# 3(antisense) | UUGAGCCGAAGUCCAGGAG |
| siSP1# 1(sense) | CCUGGAGUGAUGCCUAAUATT |
| siSP1# 1(antisense) | UAUUAGGCAUCACUCCAGGTT |
| siSP1# 2(sense) | GUGCAAACCAACAGAUUAUTT |
| siSP1# 2(antisense) | AUAAUCUGUUGGUUUGCACTT |

**Supplementary Table 4. Antibodies information in this study.**

| | | Western blot | IHC |
|---|---|---|---|
| YAP1 Rabbit Polyclonal Antibody | Proteintech#13584-1-AP | 1:5000-1:50000 | 1:50-1:500 |
| SP1(D4C3) Rabbit mAb | Cell Signaling Technology#9389 | 1:1000 | 1:2000 |
| Ki67 Rabbit Polyclonal antibody | Proteintech #27309-1-AP | | 1:4000 |
| Caspase 3 Rabbit Polyclonal antibody | Proteintech#19677-1-AP | 1:500-1:1000 | |
| PARP1 Rabbit Polyclonal antibody | Proteintech#13371-1-AP | 1:500-1:2000 | |
| BCL2 Rabbit Polyclonal antibody | Proteintech#12789-1-AP | 1:1000-1:4000 | |
| Cyclin D1 Mouse Monoclonal antibody | Proteintech#60186-1-lg | 1:2000-1:10000 | |
| CTGF Rabbit Polyclonal antibody | Proteintech#23936-1-AP | 1:500-1:1000 | |
| SURVIVIN(BIRC5) Rabbit Polyclonal antibody | Proteintech#10508-1-AP | 1:500-1:2000 | |
| HRP-conjugated GAPDH Mouse Monoclonal antibody | Proteintech#HRP-60004 | 1:5000-1:50000 | |
| GAPDH Rabbit Polyclonal antibody | Proteintech#10494-1-AP | 1:10000-1:40000 | |
| Goat Anti-Mouse IgG antibody | Bioss#bs-0296G | 1:5000 | |
| Goat Anti-rabbit IgG antibody | Bioss#bs-0295G | 1:5000 | |

**Supplementary Table 5. Clinical characteristics of CRC patients who received 5FU-based chemotherapeutic treatment.**

| Patient(S*/R*) | Age | Gender(F*/M*) | Pathological diagnosis | TNM | Weeks of treatment |
|---|---|---|---|---|---|
| S1 | 54 | F | CRC | $T_3N_1M_1$ | 24 |
| S2 | 52 | M | CRC | $T_2N_2M_0$ | 21 |
| S3 | 36 | F | CRC | $T_2N_0M_0$ | 24 |
| S4 | 36 | M | CRC | $T_3N_1M_0$ | 16 |
| S5 | 58 | M | CRC | $T_3N_1M_0$ | 22 |
| S6 | 63 | F | CRC | $T_2N_1M_0$ | 15 |
| S7 | 51 | M | CRC | $T_3N_2M_0$ | 16 |
| S8 | 71 | F | CRC | $T_3N_1M_0$ | 16 |
| S9 | 42 | M | CRC | $T_2N_0M_0$ | 19 |
| S10 | 34 | F | CRC | $T_2N_2M_0$ | 22 |
| S11 | 39 | F | CRC | $T_3N_1M_0$ | 21 |
| S12 | 67 | F | CRC | $T_3N_1M_1$ | 15 |
| S13 | 49 | M | CRC | $T_3N_1M_0$ | 12 |
| S14 | 59 | F | CRC | $T_2N_1M_0$ | 23 |
| S15 | 44 | F | CRC | $T_2N_1M_0$ | 21 |
| S16 | 47 | M | CRC | $T_3N_0M_0$ | 13 |
| S17 | 56 | M | CRC | $T_1N_0M_0$ | 13 |
| S18 | 48 | F | CRC | $T_3N_1M_0$ | 21 |
| S19 | 63 | M | CRC | $T_2N_1M_0$ | 18 |
| S20 | 72 | F | CRC | $T_1N_0M_0$ | 21 |
| S21 | 47 | F | CRC | $T_3N_1M_1$ | 21 |
| S22 | 31 | F | CRC | $T_4N_1M_0$ | 18 |
| S23 | 47 | M | CRC | $T_2N_0M_0$ | 21 |
| S24 | 40 | M | CRC | $T_3N_0M_0$ | 24 |
| S25 | 43 | F | CRC | $T_4N_0M_0$ | 20 |
| S26 | 58 | M | CRC | $T_2N_1M_0$ | 21 |
| S27 | 72 | M | CRC | $T_2N_1M_0$ | 21 |
| S28 | 49 | F | CRC | $T_4N_1M_0$ | 24 |
| S29 | 39 | F | CRC | $T_3N_1M_0$ | 11 |
| S30 | 51 | M | CRC | $T_3N_2M_0$ | 16 |
| R1 | 63 | M | CRC | $T_2N_0M_0$ | 20 |
| R2 | 46 | M | CRC | $T_1N_1M_0$ | 21 |
| R3 | 39 | F | CRC | $T_4N_0M_0$ | 15 |
| R4 | 58 | M | CRC | $T_4N_1M_1$ | 16 |
| R5 | 57 | M | CRC | $T_2N_2M_0$ | 24 |
| R6 | 47 | M | CRC | $T_2N_1M_0$ | 15 |
| R7 | 45 | F | CRC | $T_3N_0M_0$ | 21 |
| R8 | 28 | F | CRC | $T_2N_0M_1$ | 21 |
| R9 | 37 | M | CRC | $T_3N_1M_0$ | 21 |

| | | | | | |
|---|---|---|---|---|---|
| R10 | 46 | F | CRC | $T_3N_0M_0$ | 18 |
| R11 | 57 | F | CRC | $T_4N_0M_0$ | 24 |
| R12 | 55 | M | CRC | $T_3N_1M_0$ | 24 |
| R13 | 37 | F | CRC | $T_4N_2M_1$ | 24 |
| R14 | 49 | M | CRC | $T_3N_0M_0$ | 21 |
| R15 | 51 | M | CRC | $T_2N_2M_0$ | 24 |
| R16 | 47 | F | CRC | $T_4N_2M_1$ | 21 |
| R17 | 68 | M | CRC | $T_3N_1M_0$ | 21 |
| R18 | 61 | F | CRC | $T_2N_1M_1$ | 15 |
| R19 | 59 | M | CRC | $T_3N_2M_0$ | 27 |
| R20 | 55 | M | CRC | $T_2N_1M_0$ | 22 |
| R21 | 50 | M | CRC | $T_2N_1M_0$ | 21 |
| R22 | 63 | M | CRC | $T_2N_0M_0$ | 18 |
| R23 | 46 | M | CRC | $T_1N_1M_0$ | 17 |
| R24 | 39 | F | CRC | $T_4N_0M_0$ | 21 |
| R25 | 58 | M | CRC | $T_4N_1M_1$ | 21 |
| R26 | 57 | M | CRC | $T_2N_2M_0$ | 21 |
| R27 | 66 | F | CRC | $T_3N_2M_0$ | 22 |
| R28 | 72 | M | CRC | $T_3N_2M_0$ | 21 |
| R29 | 68 | F | CRC | $T_3N_1M_0$ | 21 |
| R30 | 59 | F | CRC | $T_2N_1M_1$ | 24 |

**S*, Sensitivity, 5FU-sensitive.** Patients who responded well to 5FU-based chemotherapeutic treatment;
**R*, Relapse, 5FU-resistant.** Patients who relapsed after the first 5FU-based chemotherapeutic treatment.
**F*,** Female. **M*,** Male.
